# *Damnacanthus giganteus* extract block diffuse large b-cell lymphoma proliferation and EMT by regulating mitochondrial dysfunction and glycolysis

**DOI:** 10.1186/s41065-025-00531-3

**Published:** 2025-08-25

**Authors:** Peng Yang, GuangYun Zhou, XiuMei Ma, MingTao Huang, ZuJie Qin, Bing Qing, SuLing Chen, JiangCun Wei

**Affiliations:** 1 Science and Technology Dept, Guangxi International Zhuang Medicine Hospital Affiliated to Guangxi University of Chinese Medicine, Guangxi Zhuang Autonomous Region, No. 8 Qiuyue Road, Wuxiang New District,, Nanning City, 530201 China; 2 Graduate School of Guangxi, Guangxi Zhuang Autonomous Region, University of Chinese Medicine, Nanning City, 530222 China; 3 Department of Pediatrics, Guangxi International Zhuang Medicine Hospital, Guangxi University of Chinese Medicine, Zhuang Autonomous Region, Nanning City, 530201 Guangxi China; 4 Zhuang-Yao Medicine Preparation Center, Guangxi International Zhuang Medicine Hospital Affiliated to Guangxi University of Chinese Medicine, Guangxi Zhuang Autonomous Region, No. 8 Qiuyue Road, Wuxiang New District, Nanning City, 530201 China; 5 College of Pharmacy, Guangxi Zhuang Autonomous Region, Guangxi University of Chinese Medicine, Nanning City, 530200 China

**Keywords:** Diffuse large b-cell lymphoma, *Damnacanthus giganteus* extract, Epithelial-to-mesenchymal transition, Proliferation, Mitochondrial dysfunction, Glycolysis

## Abstract

**Background:**

In non-Hodgkin’s lymphoma, diffuse large b-cell lymphoma (DLBCL) is one of the most prevalent and commonly diagnosed subtypes. There is a need to develop more effective drugs since the currently approved drugs still have limitations.

**Methods:**

DLBCL cell lines were intervened with different concentrations of *Damnacanthus giganteus* extract (DGE). The malignant phenotype of DLBCL cells was detected by CCK-8, colony formation assay, AnnexinV-PI double staining assay, and Transwell. The effect of DGE on the in vivo growth of DLBCL cells was assessed by nude mice transplantation tumor assay and immunohistochemistry. Cellular mitochondrial function was assessed by measuring mitochondrial ROS levels, MMP, and ATP production, and glycolysis was assessed by determining glucose uptake and lactate production. The changes of epithelial-mesenchymal transition (EMT) markers were evaluated via Western blot.

**Results:**

Intervention with low-toxicity concentrations of DGE significantly inhibited proliferative capacity and clonogenic potential in DLBCL cells while concurrently enhancing apoptosis and cisplatin sensitivity. DGE treatment also suppressed migratory and invasive behaviors, accompanied by downregulation of mesenchymal markers N-cadherin and Vimentin. In vivo studies confirmed therapeutic efficacy, with DGE monotherapy showing marked tumor growth suppression and synergistic activity with cisplatin. Mechanistically, DGE exacerbated mitochondrial dysfunction and suppressed glycolysis. This mitochondrial impairment phenotype elicited by DGE treatment was recapitulated using the mitochondrial complex I inhibitor Rotenone, which similarly induced proliferation inhibition and EMT modulation. Furthermore, the ROS scavenger N-acetylcysteine partially rescued DGE-induced cellular alterations, including proliferation and EMT suppression.

**Conclusion:**

‌Our study demonstrates for the first time that DGE effectively suppresses tumor proliferation and EMT during DLBCL progression. These antitumor effects appear to be mediated through modulation of mitochondrial function and glycolysis. These findings position DGE as a promising novel therapeutic candidate for DLBCL treatment, meriting immediate clinical translation and further evaluation.

**Supplementary Information:**

The online version contains supplementary material available at 10.1186/s41065-025-00531-3.

## Introduction

Lymphomas can originate in any organ and tissue of the body with diverse clinical manifestations. Lymphomas fall into two categories: Hodgkin’s lymphoma and non-Hodgkin’s lymphomas (NHL) [1]. NHL refers to highly heterogeneous tumors originating in the lymph nodes and lymphoid tissues, the cause of which is unknown, with susceptibility factors including immunodeficiency, infections, and demographic factors [2, 3]. Globally, the incidence of NHL has increased rapidly over the past few decades [4]. Diffuse large B-cell lymphoma (DLBCL) is a malignant proliferative disease derived from mature B-cells with significant heterogeneity in molecular pathology, immunophenotype, and clinical phenotype, accounting for 30–35% of all NHL cases [5, 6]. Immunochemotherapy based on rituximab, cyclophosphamide, doxorubicin, vincristine, and prednisone is the mainstay of treatment for DLBCL, with more than 60% of patients responding effectively to treatment [7], but up to 50% of patients experience refraction or relapse after treatment, resulting in a poor prognosis [8]. DLBCL patients, therefore, require new therapeutic approaches to improve clinical efficacy and prognosis.

Anticancer drugs are being developed from natural medicines with complex elements and biological properties [9, 10]. TCM has been used in China for thousands of years in cancer treatment and adjuvant therapy, not only for treating symptoms and improving quality of life, but also for controlling tumor size and prolonging survival [11, 12]. *Damnacanthus giganteus* extract (DGE), obtained from the dried roots of *Damnacanthus giganteus* (Mak.) Nakai, has been traditionally utilized by China’s Yao ethnic group for its sedative, analgesic, anti-inflammatory, and antispasmodic properties. Current phytochemical analyses identify rubiadin-1-methyl ether, subspinosin, 8-hydroxysubspinosin, 5-hydroxydamnacanthol-ω-ethyl ether, and 8-hydroxydamnacanthol-ω-ethyl ether as DGE’s primary constituents. While existing research on *Damnacanthus giganteus* (DG) remains limited, its major anthraquinone components exhibit documented pharmacological activities, including antitumor, anti-aging, sedative, neuroprotective, and anti-inflammatory effects [13, 14]. Modern studies also report antitumor activities in related Rubiaceae species, such as *Damnacanthus indicus* and *Damnacanthus officinarum* [15, 16]. However, the efficacy of DG cannot be inferred from that of its congeners. Therefore, the present research aims to provide the first evidence of DG’s therapeutic potential and underlying mechanisms against DLBCL.

The present study demonstrated the tumorsuppressive effects of DGE on DLBCL cells, and its mechanism likely involves modulation of mitochondrial function and glycolysis. These findings not only provide direct evidence for the anticancer potential of DGE but also expand therapeutic options for DLBCL patients.

## Materials and methods

### Preparation of DGE

*Damnacanthus giganteus* was purchased from Gongcheng County, Guangxi, and was identified as the dried root of *Damnacanthus giganteus* (Mak.) Nakai. *Damnacanthus giganteus* (1000 g) was soaked in 10 times water for 1/2 h and boiled for 1 h. Then, *Damnacanthus giganteus* was boiled in water at an 8:1 ratio for one hour, a process repeated twice. The three filtrates obtained were mixed and concentrated. After that, the decoction was extracted with 95% ethanol, filtered and concentrated into extract, fully solubilized with distilled water, formulated into the required concentration, and stored at -20℃.

### Cell culture and treatment

Normal human B lymphocytes (GM12878) with human DLBCL cell line SU-DHL4 (ATCC, VA, USA) were placed in Iscove’s modifed Dulbecco’s medium (Gibco, Grand Island, NY, USA) containing 10% fetal bovine serum (FBS, Gibco) and penicillin/streptomycin 100 U/mL and cultured at 37 °C, 5% CO_2_. Every other day, the medium was changed, and logarithmic growth phase cells were harvested.

### Cell treatment

Cells were treated with different concentrations of DGE (25, 50, 100, 200 and 400 µg/mL) and cultured for 24 h at 37 ℃ in a constant temperature incubator.

As a positive control, doxorubicin (DOX; MedChemExpress, New Jersey, USA) was employed. This first-line therapeutic agent for DLBCL exerts cytotoxic effects through multiple mechanisms: DNA intercalation, inhibition of topoisomerase II, and reactive oxygen species generation, ultimately inducing DNA damage and cell death [17]. DLBCL cells were treated with different concentrations of doxorubicin (0–1 µM) and cultured for 24 h at 37 ℃ in a constant temperature incubator.

To investigate the role of mitochondrial dysfunction, cells were treated with a mitochondrial complex I inhibitor, Rotenone (Rot, 0.5 µM; Sigma-Aldrich, St. Louis, MO, USA) or with a combination of 100 µg/mL of DGE and 20 mM of the ROS scavenger N-acetylcysteine (NAC, Sigma-Aldrich) for 24 h. The administration protocols for Rot and NAC were carefully optimized through preliminary dose-response experiments.

### CCK-8

GM12878 cells and SU-DHL4 cells were washed with PBS solution 2 times and digested with 0.25% trypsin. Cells were resuspended by adding a small amount of medium, diluted to 20 cells/µl, and plated in a 96-well plate (2 × 10^3^ cells/well). After 24 h of cell intervention, 10 µl of CCK-8 was added to each well, and incubation was continued for 3 h. The absorbance was detected at 450 nm. The IC50 value was calculated according to the dose-response curve.

### EdU

Cells were plated into 96-well plates at 6000 cells/well, and continued to incubate for 24 h. As long as 60% confluence, cells in each well were covered with 100 µL EdU solution (Ribobio, Guangzhou, China) for 2 h. Fluorescence microscopy was performed for observation and photographs were taken.

### Colony formation experiment

Cells, after trypsin digestion, were fully dispersed at 500 cells/well in 6-well plates and placed at 37℃, 5% CO_2_ for 14 days. After that, cells were methanol-fixed for 30 min, stained with 0.1% crystalline violet, and photographed.

### AnnexinV-PI double staining

Cell digestion was performed with 0.25% Trypsin (EDTA-free) (Boster, Wuhan, China), followed by centrifugation twice, with the supernatant removed each time. Annexin-V-FITC/PI staining solution was formulated using the Annexin-V-FITC apoptosis detection kit (ApexBio, TX, USA), and cells were resuspended (1 × 10⁶ cells per 100 µL staining solution). After staining for 15 min, apoptosis was detected by flow cytometry (BD Biosciences, NJ, USA).

### Transwell

For detecting migration, Transwell chamber (Corning, Steuben County, NY, USA) was utilized, and Matrigel was coated on the Transwell chamber for determining cell invasive ability. Cells were digested, washed twice with serum-free culture medium, resuspended and counted. The cell suspension (200 µL, 1 × 10^5^) was added in the upper Trasnwell chamber, with 500 µL DMEM medium containing 10% FBS (20 ng/ml) in the lower chamber. After 24 h, the cells that appeared in the lower chamber were fixed with 4% paraformaldehyde for 10 min and stained with crystal violet. The cell staining was photographed under a light microscope, and cell counting was performed using ImageJ software (NIH, MD, USA).

### Mitochondrial ROS

Mitochondrial ROS were detected using MitoSOX Red (ThermoFisher Scientific, Waltham, MA, USA). Cells were incubated with MitoSOX Red at 37 °C for 30 min, followed by observation of red fluorescence (excitation/emission wavelengths: 396 nm/610 nm) under a laser scanning confocal microscope. Finally, the relative fluorescence intensity was calculated using ImageJ software. Higher intensity of red fluorescence indicates higher mitochondrial ROS levels, and vice versa, lower mitochondrial ROS levels.

### Mitochondrial membrane potential (MMP) assay

The cells were centrifuged twice, with the supernatants discarded each time. The cells were stained with reference to the JC-1 MMP Detection Kit (Beyotime, Shanghai, China), and the red/green fluorescence changes were detected on a flow cytometer.

### ATP assay

In the 96-well plate, cell supernatant samples were mixed with 20 µL ATP standard solution and 100 µLATP assay working solution (ATP assay kit; Beyotime) in 96-well plate. RLU values were measured by chemiluminescence, and ATP level was calculated according to the standard curve.

### Glucose uptake and lactate production

Glucose uptake assay was performed using Screen QuestTM colourimetric glucose uptake assay kit (Nanjing Jiancheng Bioengineering Institute, Jiangsu, China). The absorbance ratio was measured at 570 nm/610 nm using a fluorescence microplate reader. Lactic acid production was detected by lactic acid assay kit (Nanjing Jiancheng Bioengineering Institute). Optical density (OD) was measured at 530 nm. The procedures were executed as per the kit instructions, respectively.

### Western blot

The collection of proteins was achieved using RIPA lysis buffer (Beyotime). and protein was determined using the BCA technique. After extraction, the protein and sampling buffer were combined in a 2:1 ratio, boiled and denatured, and separated using SDS-PAGE. The protein was transferred to the polyvinylidene fluoride membrane, detected with the primary antibodies against E-cadherin (1:1000, #3195, Cell Signaling Technology, USA), N-cadherin (1:1000, #13116, Cell Signaling Technology), Vimentin (1:1000, #5741, Cell Signaling Technology), PTEN-induced kinase 1 (PINK1; 1:1000, ab216144, Abcam, USA), Parkin RBR E3 ubiquitin protein ligase (PRKN/Parkin; 1:1000, #2132, Cell Signaling Technology) and β-actin (1:1000, #4967, Cell Signaling Technology) overnight, and combined with the corresponding horseradish peroxidase-labeled secondary antibody (1:5000, Abcam) for 1 h. After ECL exposure, target proteins were calculated using ImageJ software (NIH, MD, USA).

### In vivo assay

A group of twenty BALB/C female nude mice, weighing between 16 and 20 g and aged 6 weeks, acquired from Shanghai SLAC Laboratory Animals, resided in an SPF setting with a humidity of 55 ± 5% and a temperature range of 20–22 °C. The experiment was approved and reviewed by Guangxi University of Chinese Medicine Animal Ethics Committee (No. DW20240919-197). Following a week of acclimatization feeding, 0.1 mL PBS (with 1 × 10^7^ cells) was injected subcutaneously into each mouse’s axilla. All mice were allocated into four groups (*n* = 3) when tumors reached an approximate average volume of 100mm^3^ (after 14 days of inoculation). The groups were as follows: DGE group (Mice were gavaged at 100 mg/kg DGE once a day), DOX group (Mice were intraperitoneally injected with 1.5 mg/kg doxorubicin once a week), DGE + DOX group (Mice gavaged at 100 mg/kg DGE once a day and intraperitoneally injected with 1.5 mg/kg doxorubicin once a week), Control group (Mice were given an equal volume amount of vehicle as control). Measurements of the tumor’s long and short diameters were taken, with volume determined as (long diameter × short diameter^2^ × 0.52). Following 3 weeks of treatment, the tumors were thoroughly removed from euthanized mice, with the tumors being measured and imaged using an electronic scale.

The tumor samples were preserved in 4% paraformaldehyde, dehydrated, encased in paraffin, and cut into 4 μm sections. The sections underwent dewaxing and hydration, followed by H_2_O_2_ to remove endogenous peroxidase and microwave to retrieve antigen. Then, the sections were blocked with 10% normal goat serum for 30 min and detected with Ki-67 (1:100, Abcam) and N-cadherin (1:200, Cell Signaling Technology) antibodies. Five fields of view were randomly selected for taking photographs and images were analyzed using the software Image J.

### Statistical analyses

All data were processed using GraphPad Prism 9 (GraphPad Software, CA, USA), and data were expressed as mean ± standard deviation. t-tests were used for comparisons between two groups, One-Way ANOVA for comparisons across multiple groups, and Tukey’s multiple comparison test for comparisons across two groups after ANOVA. *P* < 0.05 indicated that the differences were statistically significant.

## Results

### DGE inhibits cell proliferation and promotes DOX sensitivity in DLBCL cells

**Fig. 1:**
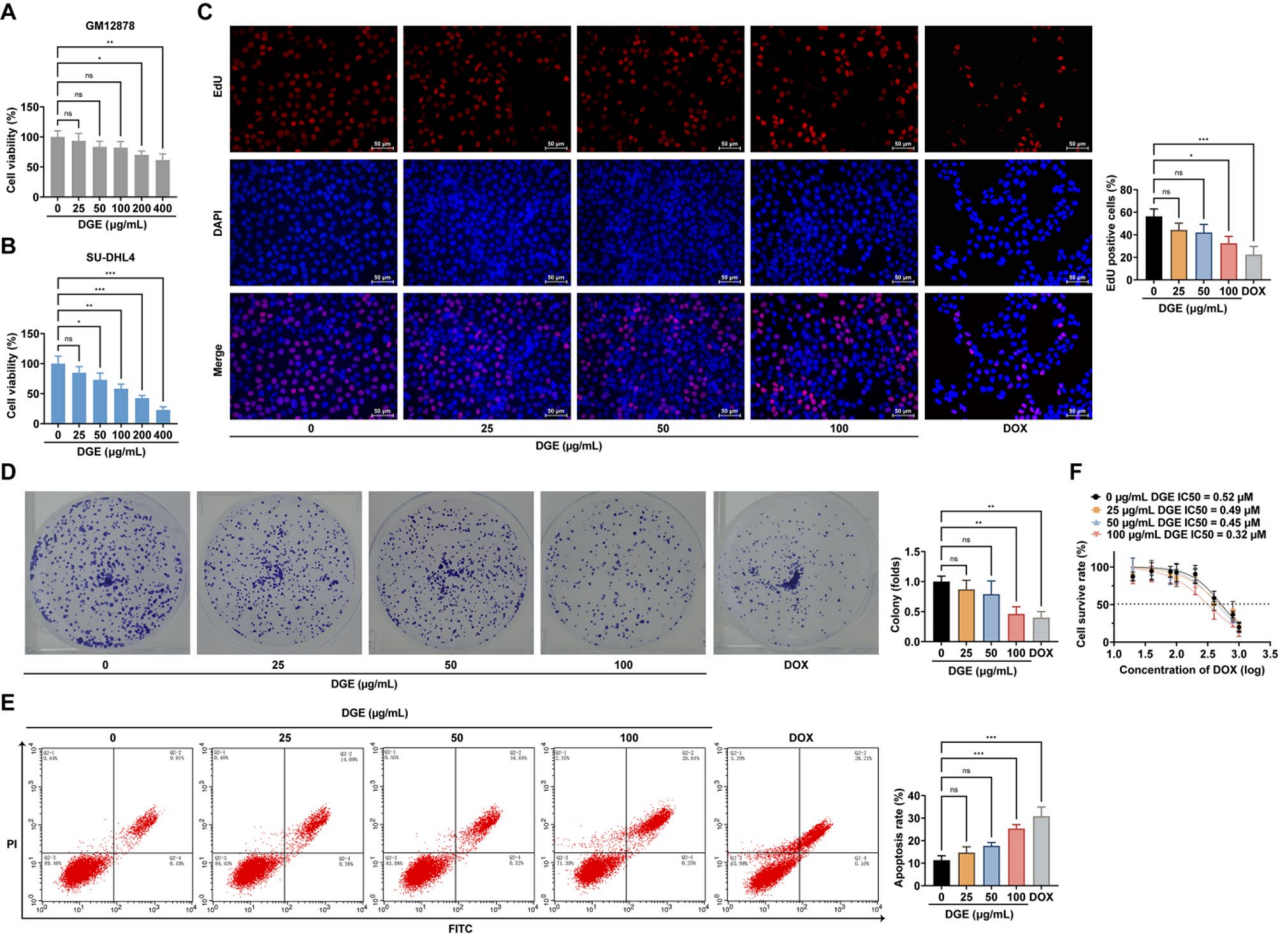
DGE inhibits cell proliferation and promotes DOX sensitivity in DLBCL cells. **A**: CCK-8 assay to detect the effect of different concentrations of DGE on the viability of GM12878 cells; **B**: CCK-8 assay to detect the effect of different concentrations of DGE on the viability of SU-DHL4 cells; **C**: EdU assay to detect the effect of different concentrations of DGE on the proliferation rate of the cells; **D**: Colony formation assay to detect the effect of different concentrations of DGE on the ability of the cells to form clones; **E**: AnnexinV-PI double staining assay to detect the effect of different concentrations of DGE on the apoptosis rate of cells; **F**: CCK-8 assay to detect the effect of different concentrations of DGE on the sensitivity of SU-DHL4 cells to DOX. Cells were treated with DGE or DOX for 24 h, DOX (0.5 µM, 24 h) was used as a positive control. Each group of experiments was repeated independently 3 times, * *P* < 0.05, ** *P* < 0.01, *** *P* < 0.001

To evaluate the effects of DGE on DLBCL, we first assessed its cytotoxicity using CCK-8 assays. Treatment with increasing DGE concentrations (25–400 µg/mL) demonstrated significantly stronger growth inhibition in human DLBCL cells (SU-DHL4, with an IC50 value of 137.6 µg/mL) compared to normal human B lymphocytes (GM12878) (Fig. 1A, B). Based on this differential toxicity profile, three low-toxicity concentrations (25, 50, and 100 µg/mL) were selected for subsequent DLBCL experiments.

EdU and colony formation assays revealed that all tested DGE concentrations significantly suppressed DLBCL cell proliferation and clonogenic capacity (Fig. 1C, D). Notably, the anti-proliferative effect of 100 µg/mL DGE was comparable to that of DOX, a first-line DLBCL therapeutic included as a positive control. Further analysis via Annexin V-PI double staining demonstrated that both DGE and DOX markedly increased tumor cell apoptosis rates (Fig. 1E). Subsequent CCK-8 evaluation of DOX sensitivity showed that DGE pretreatment substantially reduced the IC50 values of DOX in DLBCL cells across gradient concentrations (Fig. 1F). Collectively, these findings indicate that DGE at low-toxicity concentrations inhibits DLBCL proliferation in a concentration-dependent manner while simultaneously enhancing cellular sensitivity to DOX chemotherapy.

### DGE impairs metastasis and EMT in DLBCL cells

**Fig. 2:**
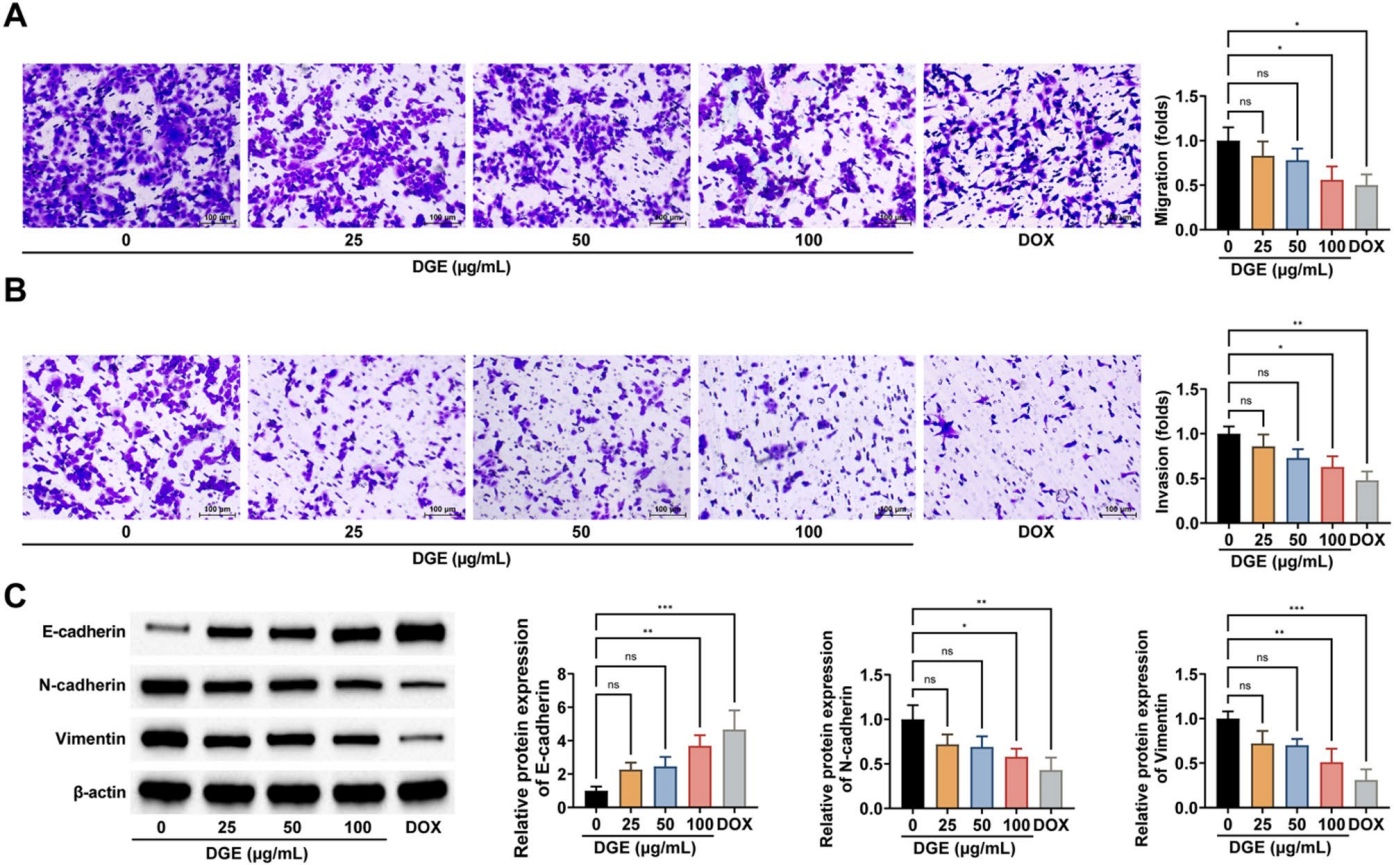
DGE impairs metastasis and EMT in DLBCL cells. **A**-**B**: Transwell assay to detect the effect of different concentrations of DGE on cell migration and invasion ability; **C**: Western blot to detect the effect of different concentrations of DGE on the expression levels of cellular E-cadherin, N-cadherin and Vimentin. Cells were treated with DGE or DOX for 24 h, DOX (0.5 µM, 24 h) was used as a positive control. Each group of experiments was repeated independently 3 times, * *P* < 0.05, ** *P* < 0.01, *** *P* < 0.001

Transwell migration and invasion assays demonstrated that both DGE and DOX treatments effectively suppressed the migratory and invasive capacities of tumor cells (Fig. 2A, B). Western blot analysis further revealed that DGE and DOX interventions downregulated mesenchymal markers (N-cadherin and Vimentin) while upregulating the epithelial marker E-cadherin, indicating suppression of EMT in tumor cells (Fig. 2C). Importantly, DGE exhibited a significant, concentration-dependent inhibitory effect on EMT progression in DLBCL cells while maintaining low cytotoxicity.

### DGE and DOX synergistically inhibit DLBCL cell growth in a mouse xenograft model

**Fig. 3:**
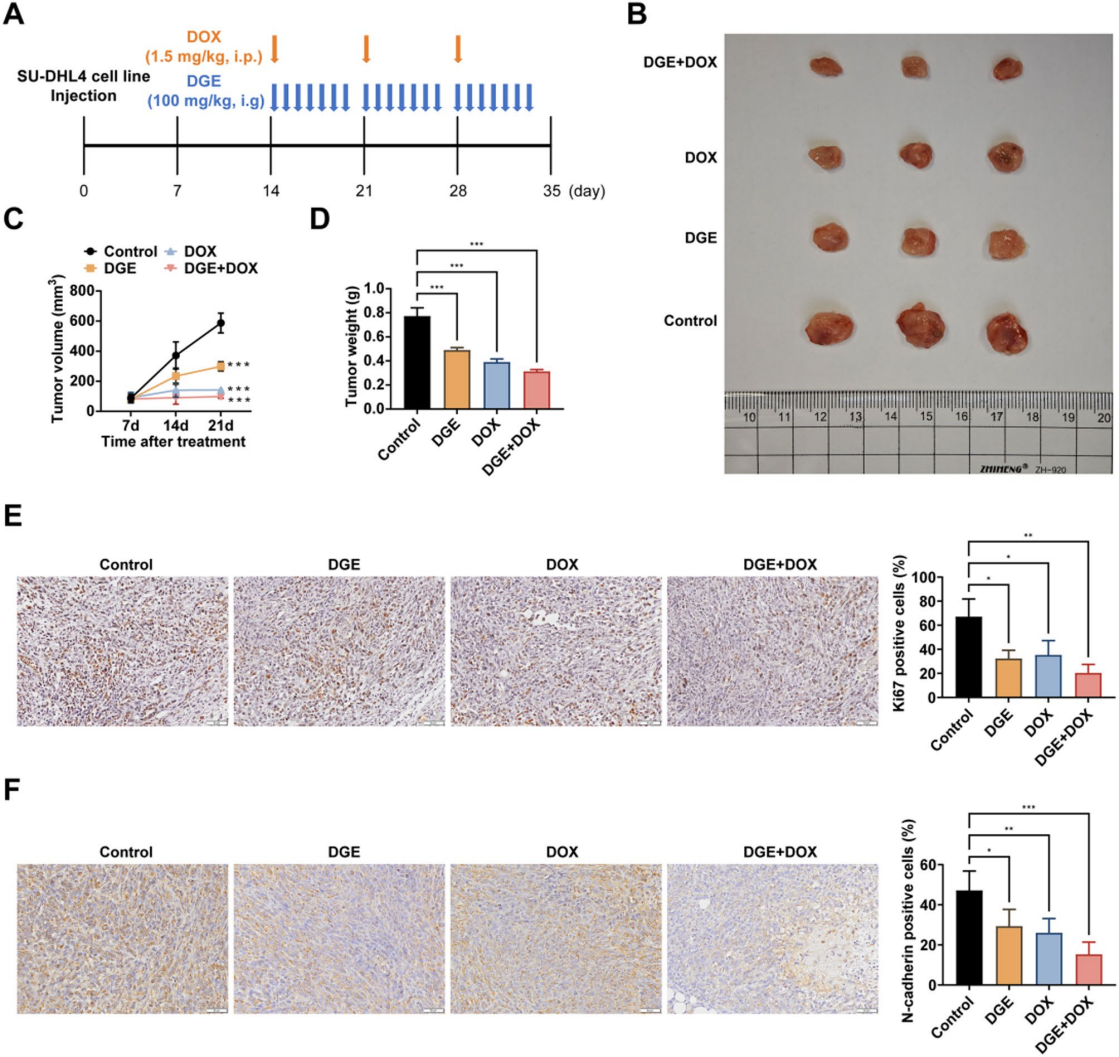
DGE and DOX synergistically inhibit DLBCL cell growth in a mouse xenograft model. **A**: Schematic diagram of the in vivo experiment. Mice bearing tumors were treated with vehicle control, DGE (100 mg/kg/once per day, i.g.), DOX (1.5 mg/kg/once per week, i.p. ), or DGE + DOX for 21 days. *n* = 3 mice per group. **B**: Representative pictures of transplanted tumors in nude mice; **C**-**D**: The volume and weight of transplanted tumors in nude mice; **E**-**F**: Immunohistochemical assessment of the positive expression levels of Ki67 and N-cadherin proteins in the tissues of transplanted tumors in nude mice. * *P* < 0.05, ** *P* < 0.01, *** *P* < 0.001

To evaluate the in vivo antitumor efficacy of DGE against DLBCL, we established a mouse xenograft model using SU-DHL4 cells (Fig. 3A). Both DGE and DOX monotherapy groups exhibited significant suppression of tumor growth and volume, consistent with our previous in vitro observations (Fig. 3B-D). Notably, the combination therapy demonstrated superior tumor growth inhibition compared to either treatment alone (Fig. 3B-D). Subsequent immunohistochemical analysis of Ki67 (proliferation marker) and N-cadherin (EMT marker) revealed that DGE potentiated DOX’s anti-DLBCL effects, as shown by decreased Ki67- and N-cadherin-positive cells (Fig. 3E, F). These in vivo findings corroborated our in vitro results, further supporting DGE’s role in inhibiting DLBCL progression through dual suppression of proliferation and EMT pathways.

### DGE regulates mitochondrial function and glycolysis in DLBCL cells

Next, to elucidate the molecular mechanisms underlying DGE’s anti-DLBCL effects, we systematically investigated its impact on mitochondrial function and metabolic reprogramming.

**Fig. 4:**
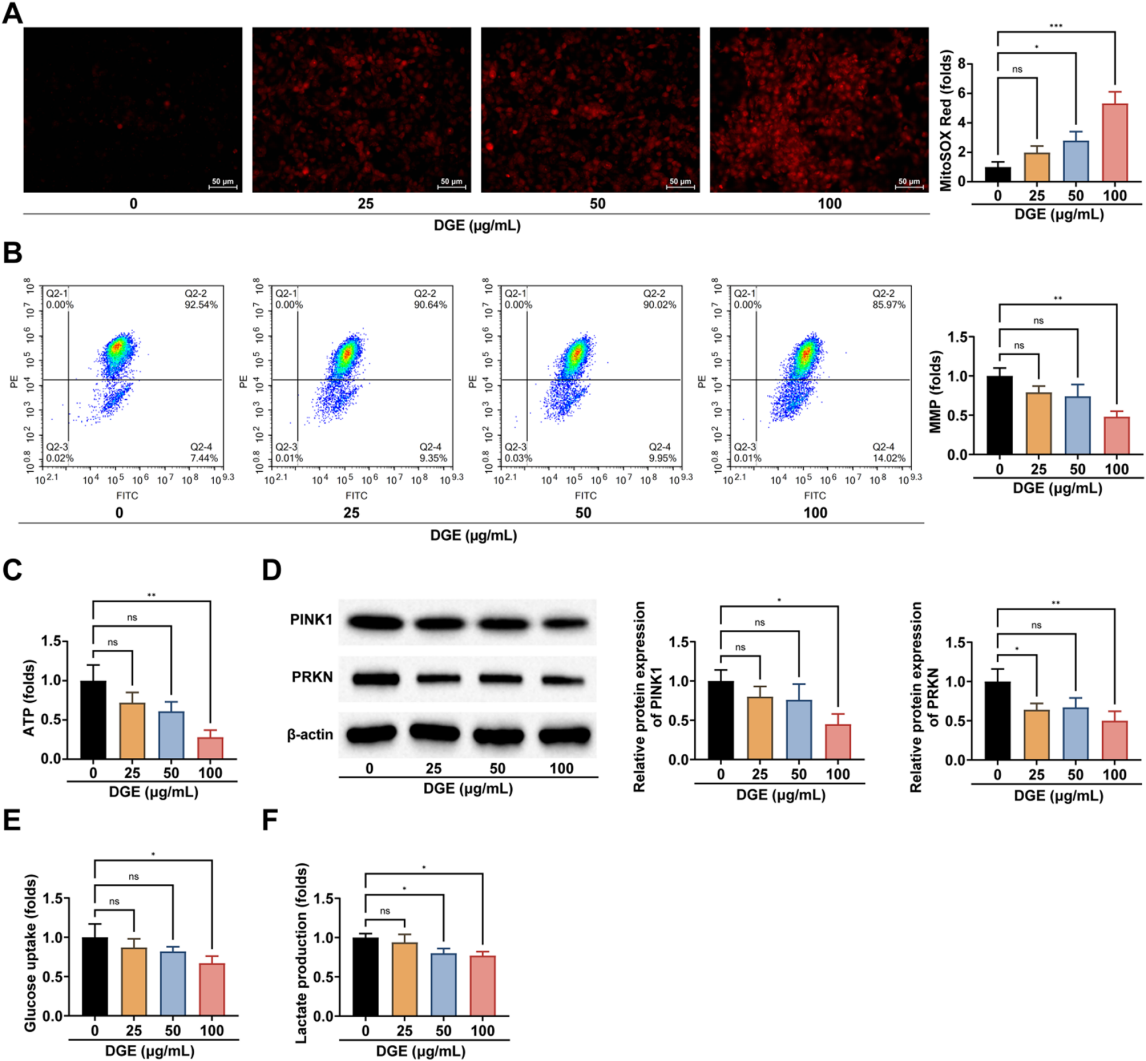
DGE regulates mitochondrial function and glycolysis in DLBCL cells. **A**: MitoSOX Red staining to assess the effect of different concentrations of DGE on mitochondrial ROS; **B**: Flow cytometry analysis of the effect of different concentrations of DGE on MMP; **C**: Kits to detect the effects of different concentrations of DGE on cellular ATP production; **D**: Western blot to detect the effect of different concentrations of DGE on the expression levels of cellular PINK1 and PRKN; **E**-**F**: Kits to detect the effects of different concentrations of DGE on cellular glucose uptake and lactate production. Cells were treated with DGE for 24 h. Each group of experiments was repeated independently 3 times, * *P* < 0.05, ** *P* < 0.01, *** *P* < 0.001

As central organelles governing cellular energetics and biosynthetic precursor supply [18, 19], mitochondria play pivotal roles in tumor progression. Our comprehensive assessment revealed that DGE treatment induced mitochondrial dysfunction through three key manifestations: elevated mitochondrial ROS levels (Fig. 4A), diminished MMP (Fig. 4B), and significantly reduced ATP production (Fig. 4C). These findings collectively demonstrate DGE’s capacity to disrupt the tricarboxylic acid cycle and oxidative phosphorylation - the core energy-generating processes in eukaryotic mitochondria. Building upon the well-established crucial role of mitophagy in mitochondrial dysfunction, we examined the effect of DGE on PINK1-PRKN pathway, a canonical mitophagy regulator. Western blot analysis confirmed a significant downregulation of both PINK1 and PRKN/Parkin expression induced by DGE treatment (Fig. 4D), suggesting impaired mitochondrial quality control.

Furthermore, DGE effectively counteracted the Warburg effect, the metabolic hallmark of cancer wherein cells preferentially utilize aerobic glycolysis despite oxygen availability [20]. This was evidenced by DGE-mediated suppression of both glucose uptake and lactate production (Fig. 4E, F), indicating its dual targeting of mitochondrial function and glycolytic metabolism in DLBCL cells.

### Mitochondrial dysfunction serves as a critical determinant in the antitumour mechanisms of DGE

**Fig. 5:**
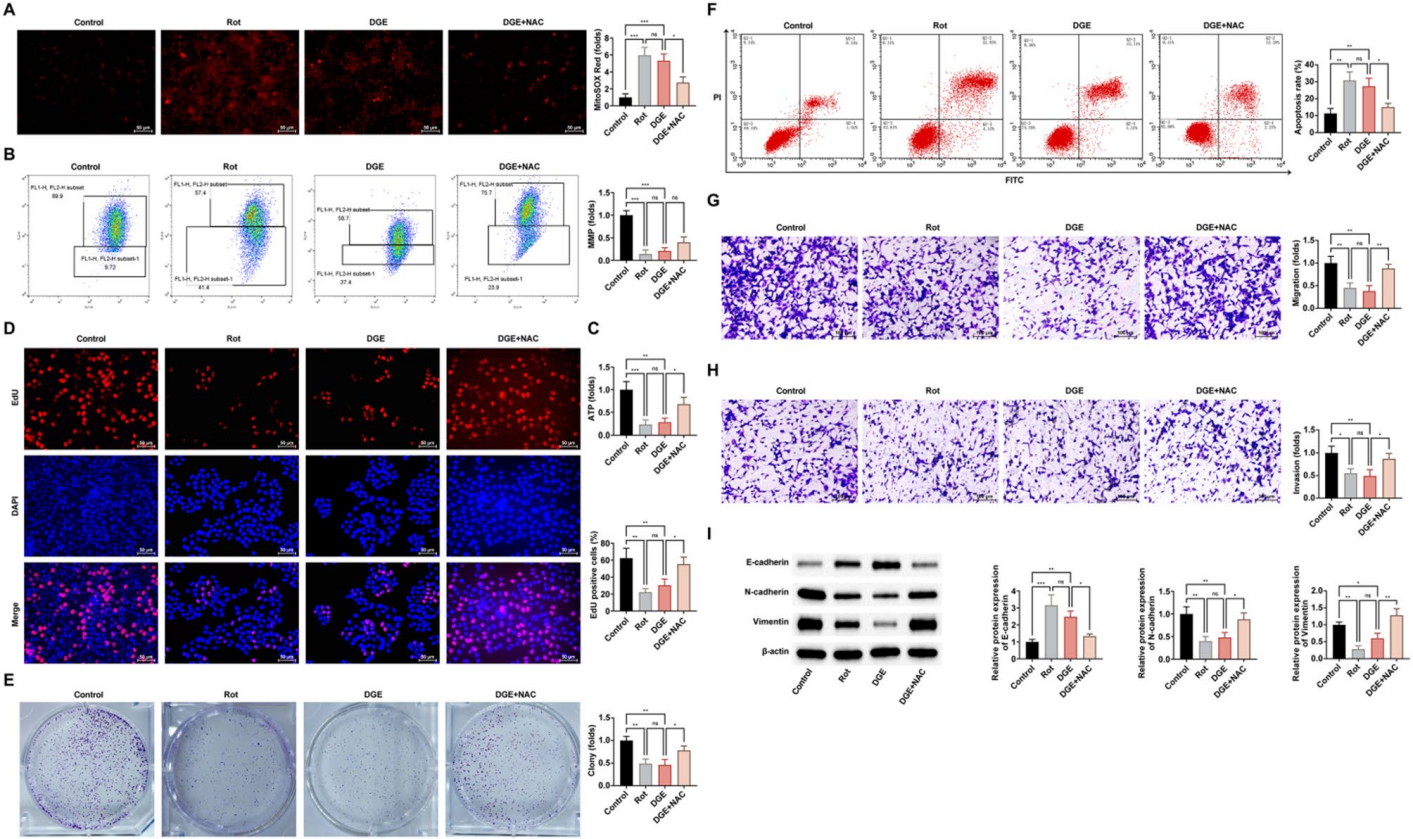
Mitochondrial dysfunction serves as a critical determinant in the antitumour mechanisms of DGE. **A**: MitoSOX Red staining to assess the cellular mitochondrial ROS levels; **B**: Flow cytometry analysis of the cellular mitochondrial membrane potential; **C**: Kit assay to detect the cellular ATP production; **D**: EdU assay to detect the cellular proliferation rate; **E**: Colony formation assay to detect the cell colony formation ability; **F**: AnnexinV-PI double staining assay to detect the cell apoptosis rate; **G**-**H**: Transwell assay to detect the migration and invasion ability of cells; **I**: Western blot to detect the expression levels of cellular E-cadherin, N-cadherin, and Vimentin. Cells were exposed to 100 µg/mL of DGE, 0.5 µM of Rot, or a combination of 100 µg/mL of DGE and 20 mM of NAC for 24 h. Each group of experiments was repeated independently 3 times, * *P* < 0.05, ** *P* < 0.01, *** *P* < 0.001

To investigate whether DGE’s suppression of malignant phenotypes in DLBCL cells stems from mitochondrial dysfunction, we employed a systematic validation strategy. Mitochondrial electron transport chain complex I inhibitor Rot was employed as a positive control. Experimental data demonstrated that Rot treatment mirrored DGE’s effects, triggering mitochondrial abnormalities characterized by elevated ROS levels, diminished MMP, and significantly reduced ATP production (Fig. 5A-C). Both interventions similarly suppressed DLBCL cell proliferation, metastasis, and EMT (Fig. 5D-I). The ROS scavenger NAC was utilized for mechanistic validation. NAC co-treatment reversed DGE-induced mitochondrial dysfunction (Fig. 5A-C). Furthermore, NAC attenuated DGE’s suppression of DLBCL cell proliferation and counteracted its inhibitory effects on migration and invasion (Fig. 5D-H). NAC also reversed DGE-mediated alterations in EMT markers by restoring N-cadherin/Vimentin expression while suppressing E-cadherin upregulation (Fig. 5I).

These collective findings demonstrate that mitochondrial dysfunction may serve as a critical mechanistic basis for DGE’s anti-tumor activity in DLBCL.

## Discussion

TCM, particularly herbal decoctions, has demonstrated notable efficacy in treating DLBCL [21, 22]. Antitumor agents derived from medicinal plants have garnered substantial attention as valuable sources of bioactive compounds. Previous studies have shown that the ethyl acetate extract obtained from *Celastrus orbiculatus Thunb.*, a historically significant medicinal plant in TCM, exhibits significant anti-neoplastic effects against non-small cell lung cancer [23]. Aqueous extracts of *Ocimum gratissimum*, possessing antifungal and antiviral properties, sensitize hepatocellular carcinoma cells to cisplatin both in vitro and in vivo [24]. Furthermore, recent reports indicate that the hydroalcoholic extract of *Tagetes erecta* exhibits cytotoxic effects in tumor cells, specifically in lung carcinoma cells, and enhances the efficacy of etoposide [25]. The present study provides the first evidence that DGE, a novel medicinal herb extract, exhibits concentration-dependent inhibition of DLBCL proliferation and suppresses EMT. Remarkably, at a concentration of 100 µg/mL, DGE demonstrated comparable antitumor efficacy to DOX, the first-line chemotherapeutic agent for DLBCL employed as our positive control. Further investigations revealed that DGE potentiates the cytotoxic effects of DOX in DLBCL cells. These in vitro findings were consistently replicated in our animal models, confirming DGE’s dual role in both attenuating DLBCL progression and synergistically enhancing DOX therapeutic efficacy.

Dysregulation of cellular metabolism is one of the emerging features of cancer. Mitochondria are important organelles responsible for many physiological processes such as energy production, metabolism, apoptosis, ROS production, calcium, and redox homeostasis [26]. Mitochondrial oxidative phosphorylation (OXPHOS) metabolic pathway provides bioenergy to tumor cells and directs macromolecular synthesis in the tumor microenvironment. Over the past century, glycolysis was once considered to be the primary metabolic mode that all tumor cells relied on to meet their energy needs. However, in recent years, more and more studies have shown that not every cancer cell’s metabolic profile is fully consistent with glycolysis, and that certain tumor subtypes rely mainly on OXPHOS rather than glycolysis for proliferation and survival [27, 28]. Mitochondrial OXPHOS and glycolysis in the cytoplasm are the two pathways for ATP production in the cell, and together they maintain the supply of ATP. Mitochondrial OXPHOS defects and ROS production are attributed to mitochondrial dysfunction [29]. Elevated levels of ROS are a common feature of cancer and can contribute to tumorigenesis by enhancing survival, proliferation, migration, invasion, and genetic instability, but also have an anti-tumor effect when ROS levels cross a threshold leading to high oxidative stress and cell death. Therefore, reaching optimal ROS levels is a driver of cancer [30]. Aggravated mitochondrial dysfunction has been shown to have anti-tumor effects in different types of cancer [31, 32], including DLBCL [33–36].

In the present study, DGE exacerbated the mitochondrial dysfunction and reduced mitochondrial aerobic glycolysis in DLBCL cells. This was evidenced by a significant increase in mitochondrial ROS levels, loss of MMP, and a marked reduction in ATP production in DLBCL cells after DGE intervention. In contrast, assessment of glycolysis levels in DLBCL cells showed that DGE intervention inhibited cellular glucose uptake and lactate production. Mitochondrial dysfunction and aerobic glycolysis have been widely accepted as hallmarks of cancer, and the interactions and mechanisms between the two during cancer development are complex. However, similar to our results, some studies in the past have also found that some of the mechanisms of anticancer therapy induce mitochondrial dysfunction while inhibiting aerobic glycolysis in cells [37, 38]. The complex nature and ambiguous phenotypic threshold of mitochondrial dysfunction complicate our understanding of its contributions to cancer [39]. Within the cellular architecture, the mitochondrial network is regulated by quality control systems, including biogenesis, dynamics, mitophagy, and proteolysis. Mitophagy, a specialized form of autophagy, is responsible for the selective degradation of aged or damaged mitochondria. This process is tightly controlled by various signaling pathways and proteins, particularly the PINK1-PRKN pathway [40]. Under physiological conditions, PINK1 translocates to the mitochondrial inner membrane for PARL (presenilin-associated rhomboid-like)-mediated cleavage and degradation. Conversely, upon mitochondrial damage or stress, the dissipation of mitochondrial membrane potential (MMP) stabilizes PINK1 on the outer mitochondrial membrane (OMM), facilitating the recruitment of cytosolic PRKN. Subsequently, this E3 ubiquitin ligase ubiquitinates OMM proteins to initiate mitophagy [41]. Recent mechanistic investigations have further illuminated the therapeutic relevance of this pathway in cancer biology [42, 43].Specifically, PINK1-mediated mitophagy has been demonstrated to sustain the survival of drug-tolerant persister cancer cells and is associated with an unfavorable prognosis [44]. Inhibition of PINK1/Parkin-dependent mitophagy enhances the sensitivity of multidrug-resistant cancer cells to B5G1, a novel betulinic acid analog [45]. The m6A methyltransferase METTL3 contributes to chemoresistance in small cell lung cancer by activating PINK1-PRKN-mediated mitophagy [46]. CDK9 inhibitors repress the SIRT1-FOXO3-BNIP3 axis and the PINK1-PRKN pathway, thereby inhibiting mitophagy initiation and promoting mitochondrial dysfunction in hepatocellular carcinoma, which disrupts mitochondrial homeostasis and induces apoptosis [47]. Furthermore, CD30, which plays a pivotal role in the growth and survival of Epstein-Barr virus (EBV)-positive DLBCL, maintains mitochondrial function in EBV-positive DLBCL cells via BNIP3-mediated mitophagy [48]. Our findings extend these observations, indicating that DGE may exert inhibitory effects on PINK1/PRKN-mediated mitophagy in DLBCL. This inhibition further exacerbates mitochondrial dysfunction and disrupts cancer cell energy metabolism, which is consistent with the observed tumor suppression. Nevertheless, it must be acknowledged that mitochondrial dysfunction engages more complex signaling networks (e.g., energy stress-AMPK/mTOR axis, calcium homeostasis, and mitochondrial unfolded protein response). Future studies are warranted to elucidate these potential mechanisms, aiming to systematically clarify the pathways and molecular processes involved in DGE-induced mitochondrial dysfunction.

Next, to elucidate the necessity of mitochondrial dysfunction in DGE-induced anticancer effects, both mitochondrial activators and inhibitors were evaluated. Rot, a canonical mitochondrial complex I inhibitor, disrupts Fe-S clusters, triggering oxidative stress, ROS accumulation, MMP collapse, ATP depletion, and subsequent cell death [49, 50]. Our data demonstrate that Rot recapitulates DGE-induced mitochondrial dysfunction and suppresses DLBCL malignant progression. Mechanistic investigations further revealed that the ROS scavenger NAC abolished DGE-mediated mitochondrial impairment and partially rescued its antitumor effects, confirming that DGE inhibits DLBCL proliferation and EMT primarily via ROS-dependent mitochondrial dysregulation. Collectively, these data underscore mitochondrial dysfunction as the central mechanism mediating DGE’s anticancer activity.

The adoption of herbal ingredients usually offers distinct advantages over conventional research models, particularly in terms of low toxicity profiles and long-term consumption safety. However, these beneficial characteristics simultaneously introduce certain methodological challenges, primarily stemming from the inherent complexity of herbal molecular compositions which complicates comprehensive mechanistic evaluations. Additionally, the current scientific literature reveals a notable knowledge gap regarding *Damnacanthus* species, with *Damnacanthus indicus* receiving limited attention and *Damnacanthus giganteus* (DG) remaining pharmacologically uncharacterized. Although current laboratory constraints and funding limitations prevent immediate experimental expansion, we have established a clear roadmap for future systematic investigations of DG’s metabolic effects. Our planned research trajectory includes detailed phytochemical profiling of DG’s active constituents, in silico prediction of bioactive compound-target interactions through computational docking approaches, and extensive pan-cancer screening using diverse cell line models to elucidate potential tumor-specific therapeutic applications. This multi-faceted strategy will address both the current knowledge gaps and the mechanistic complexities inherent in herbal medicine research.

## Conclusion

DGE inhibits DLBCL proliferation and EMT, which is related to its disruption of mitochondrial function and inhibition of glycolysis. These findings position DGE as a promising candidate for DLBCL therapy development, while simultaneously advancing the field of mitochondrial-targeted oncology interventions.

## Supplementary Information

Below is the link to the electronic supplementary material.

Supplementary Material 1

## Data Availability

The datasets used and/or analyzed during the present study are available from the corresponding author on reasonable request.
