# Supplementary figures and images for: Damnacanthus giganteus extract block diffuse large b-cell lymphoma proliferation and EMT by regulating mitochondrial dysfunction and glycolysis
Source: Hereditas. 2025 Aug 25;162:170. doi: 10.1186/s41065-025-00531-3 (PMC12379491; doi:10.1186/s41065-025-00531-3)

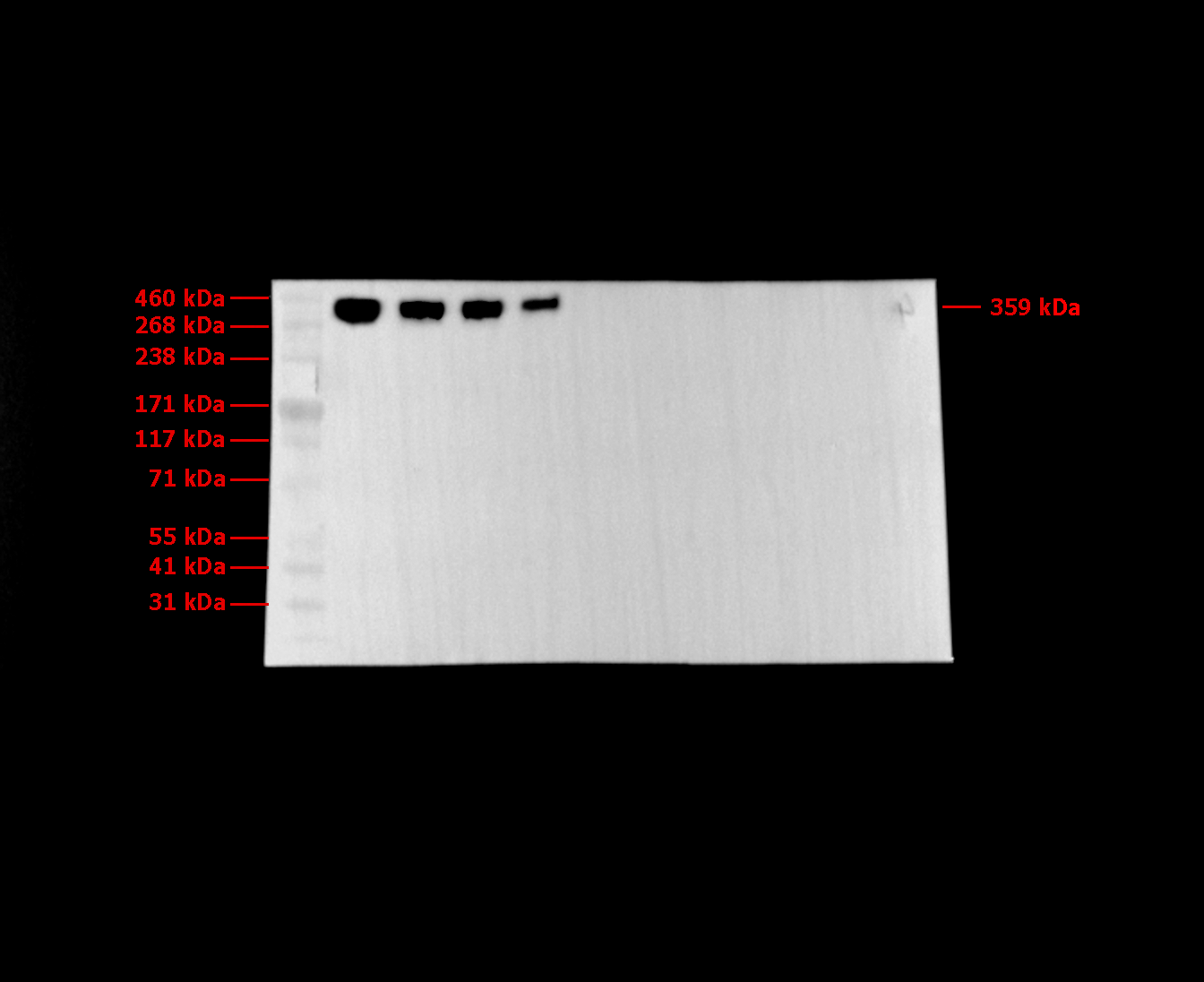

Supplement: Supplementary file 1 — Supplementary Material 1 [file 41065_2025_531_MOESM1_ESM.zip › original image for wb - marker/Original image Figure 1E/Figure 1E Ki-67.tif]

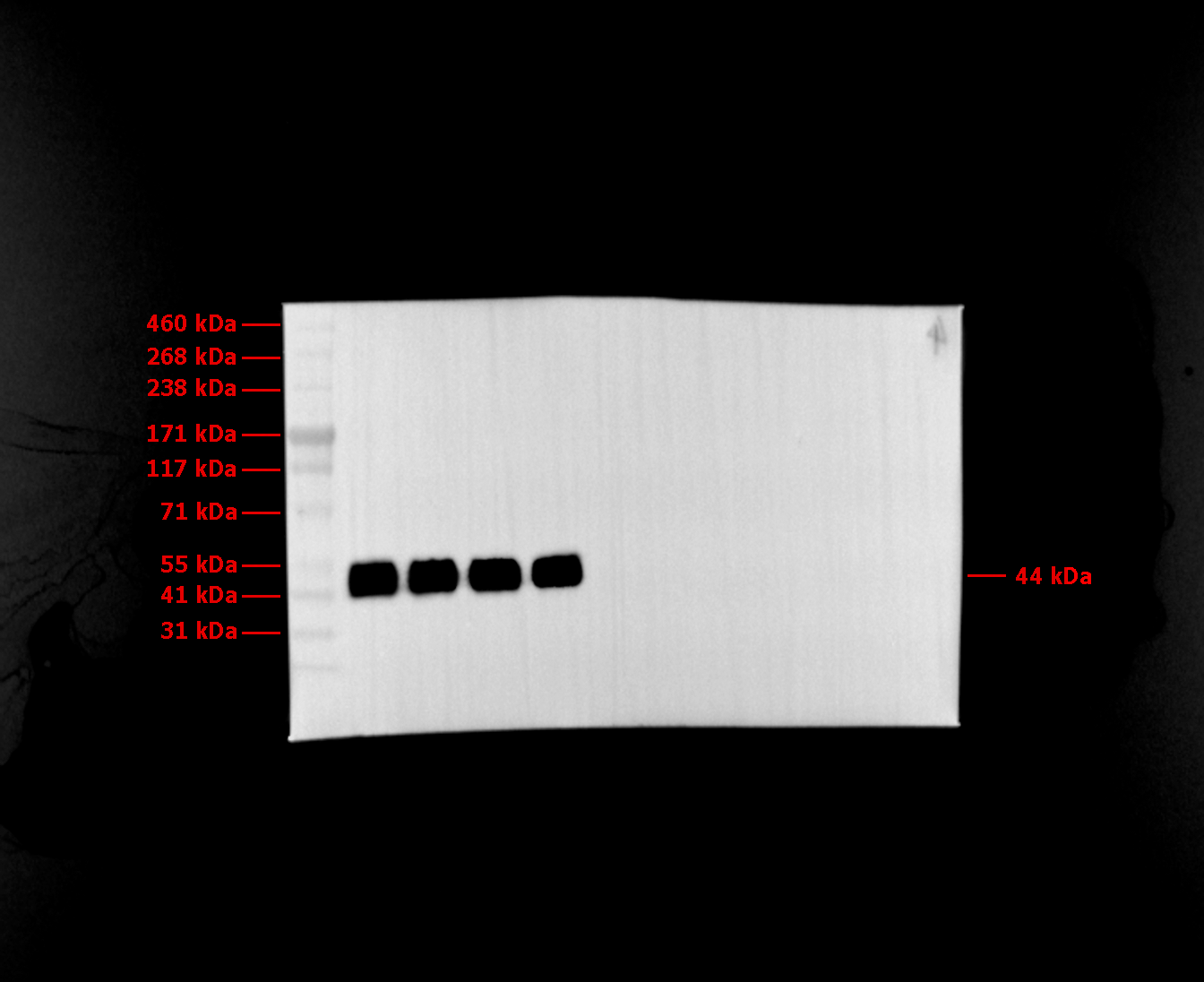

Supplement: Supplementary file 1 — Supplementary Material 1 [file 41065_2025_531_MOESM1_ESM.zip › original image for wb - marker/Original image Figure 1E/Figure 1E β-actin.tif]

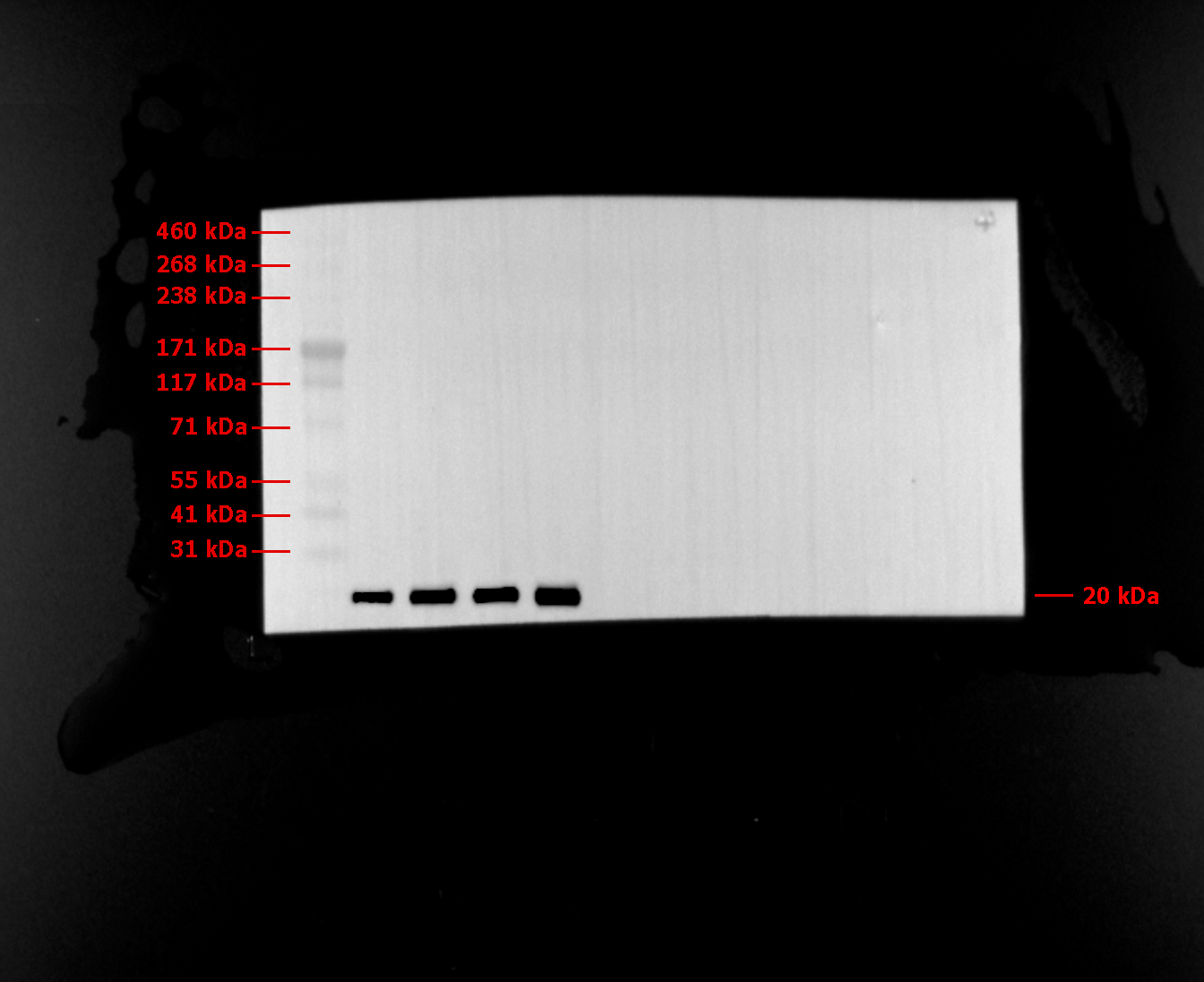

Supplement: Supplementary file 1 — Supplementary Material 1 [file 41065_2025_531_MOESM1_ESM.zip › original image for wb - marker/Original image Figure 1G/Figure 1G Bax.tif]

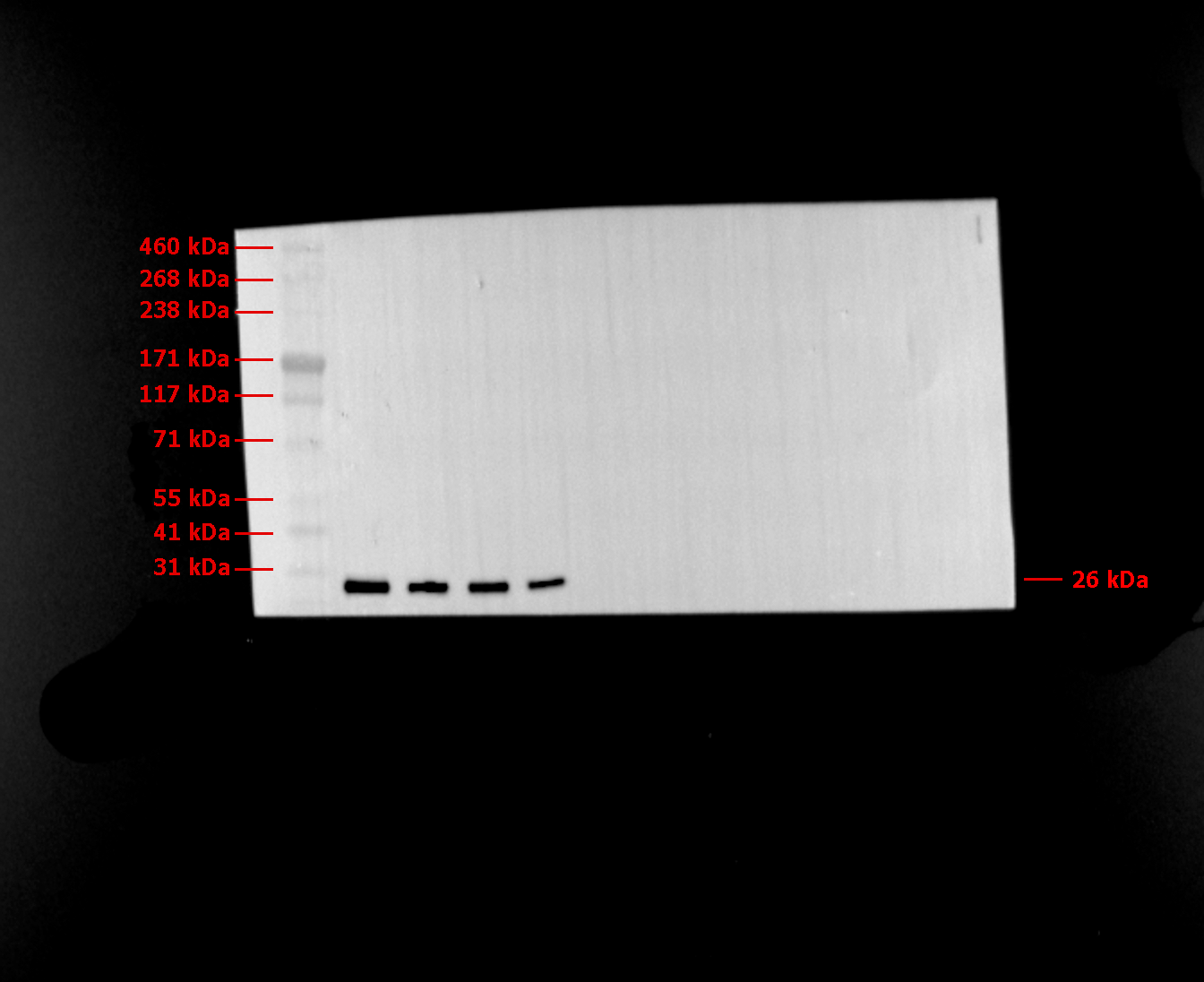

Supplement: Supplementary file 1 — Supplementary Material 1 [file 41065_2025_531_MOESM1_ESM.zip › original image for wb - marker/Original image Figure 1G/Figure 1G Bcl-2.tif]

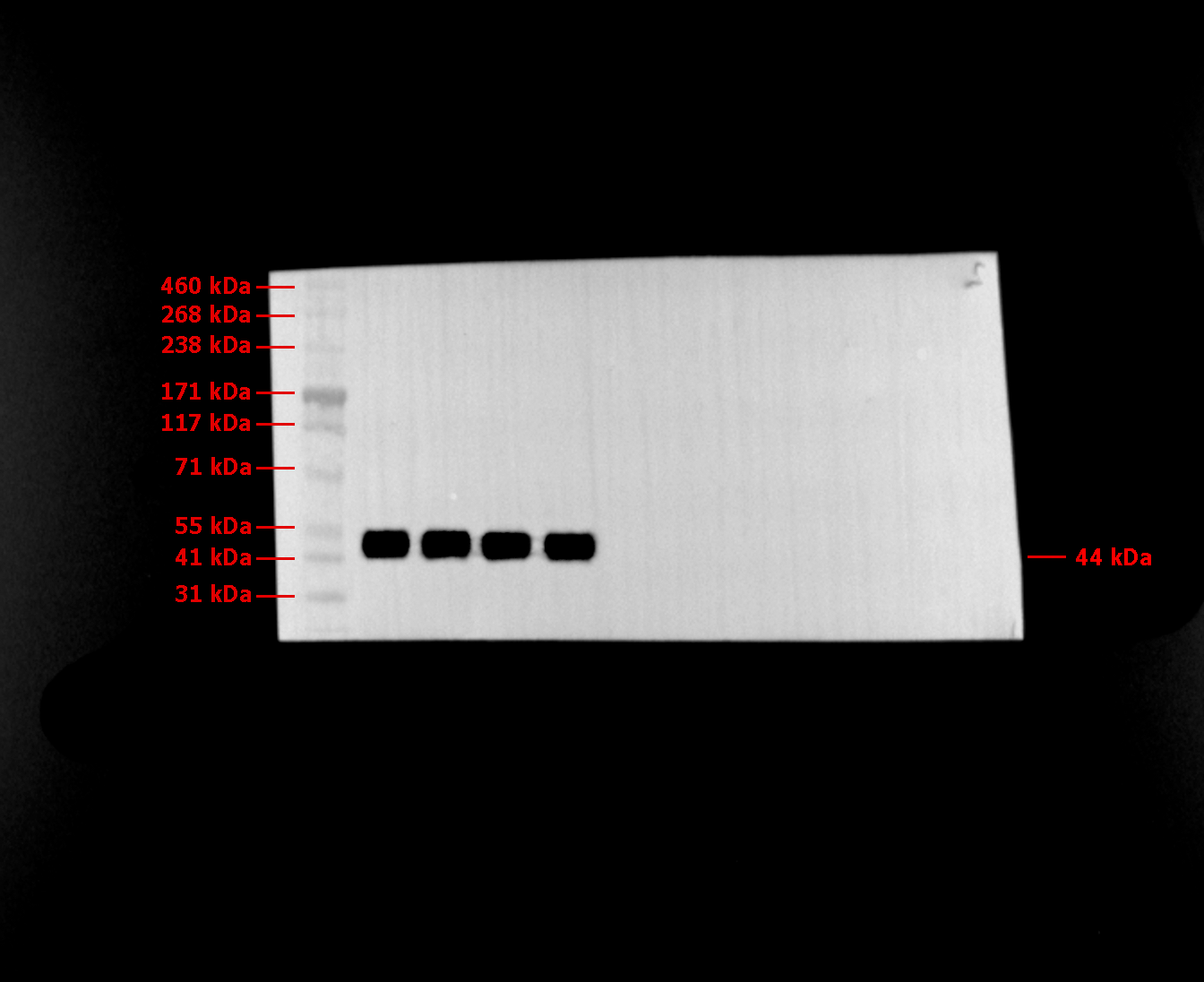

Supplement: Supplementary file 1 — Supplementary Material 1 [file 41065_2025_531_MOESM1_ESM.zip › original image for wb - marker/Original image Figure 1G/Figure 1G β-actin.tif]

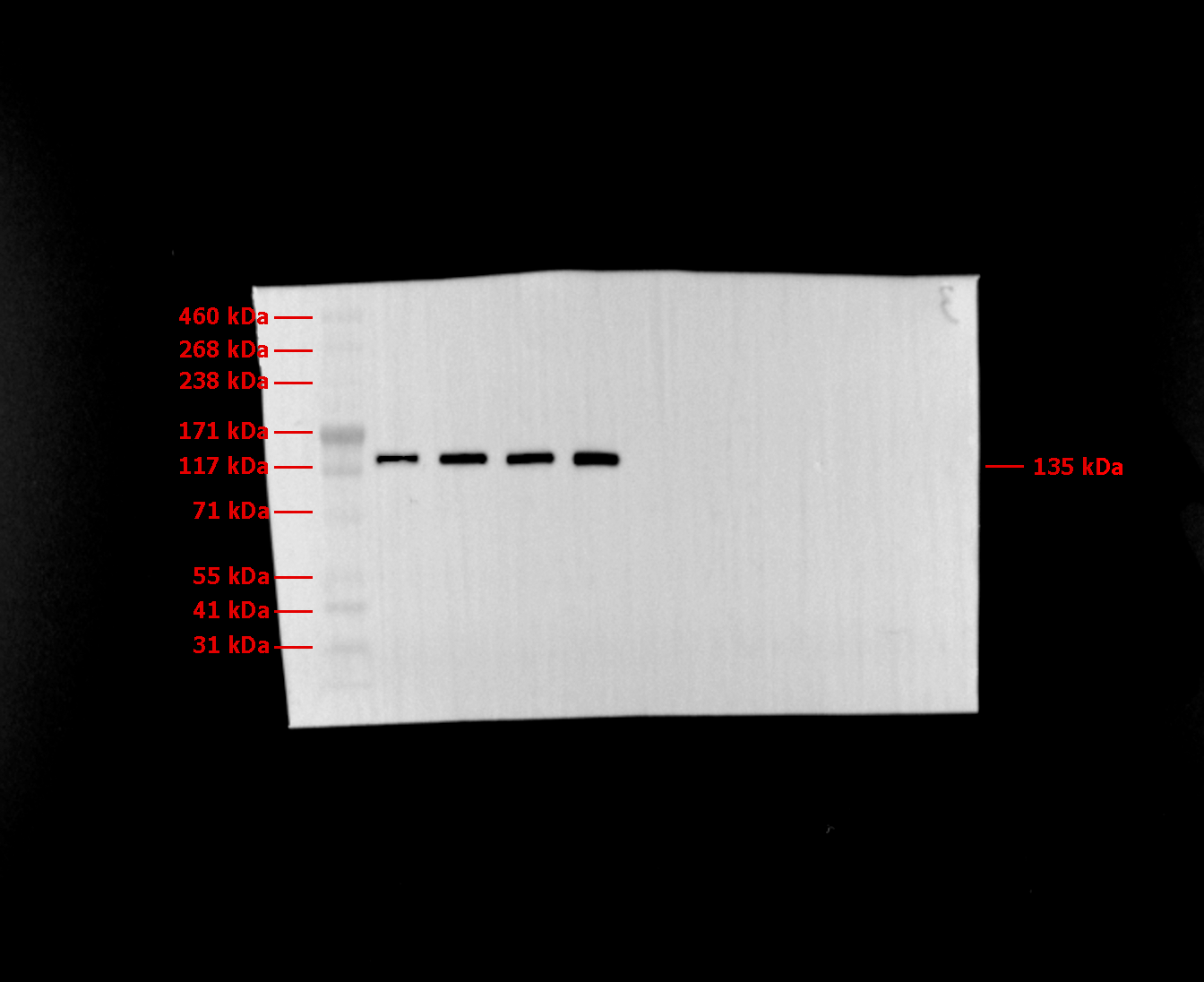

Supplement: Supplementary file 1 — Supplementary Material 1 [file 41065_2025_531_MOESM1_ESM.zip › original image for wb - marker/Original image Figure 2C/Figure 2C E-cadherin.tif]

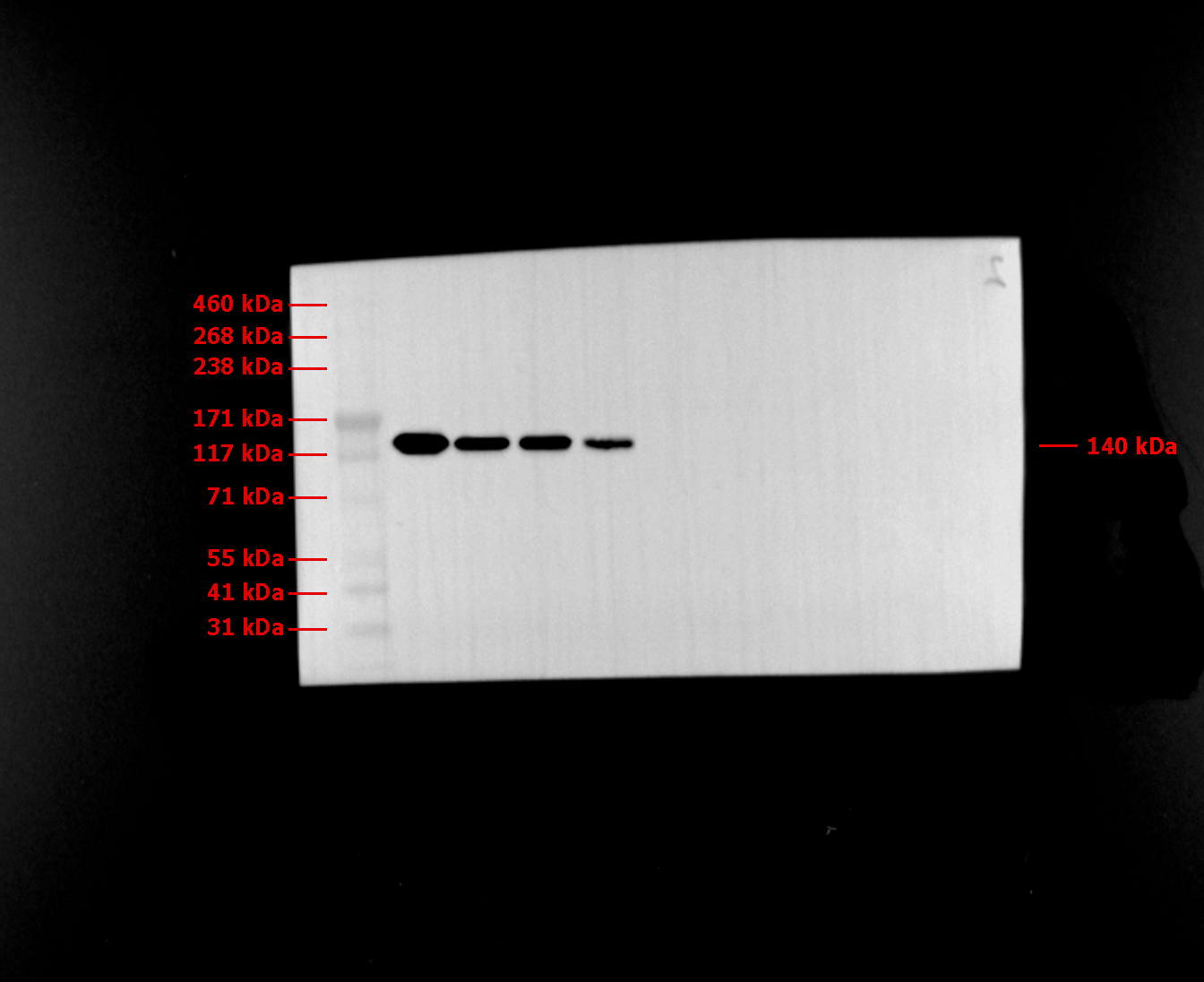

Supplement: Supplementary file 1 — Supplementary Material 1 [file 41065_2025_531_MOESM1_ESM.zip › original image for wb - marker/Original image Figure 2C/Figure 2C N-cadherin.tif]

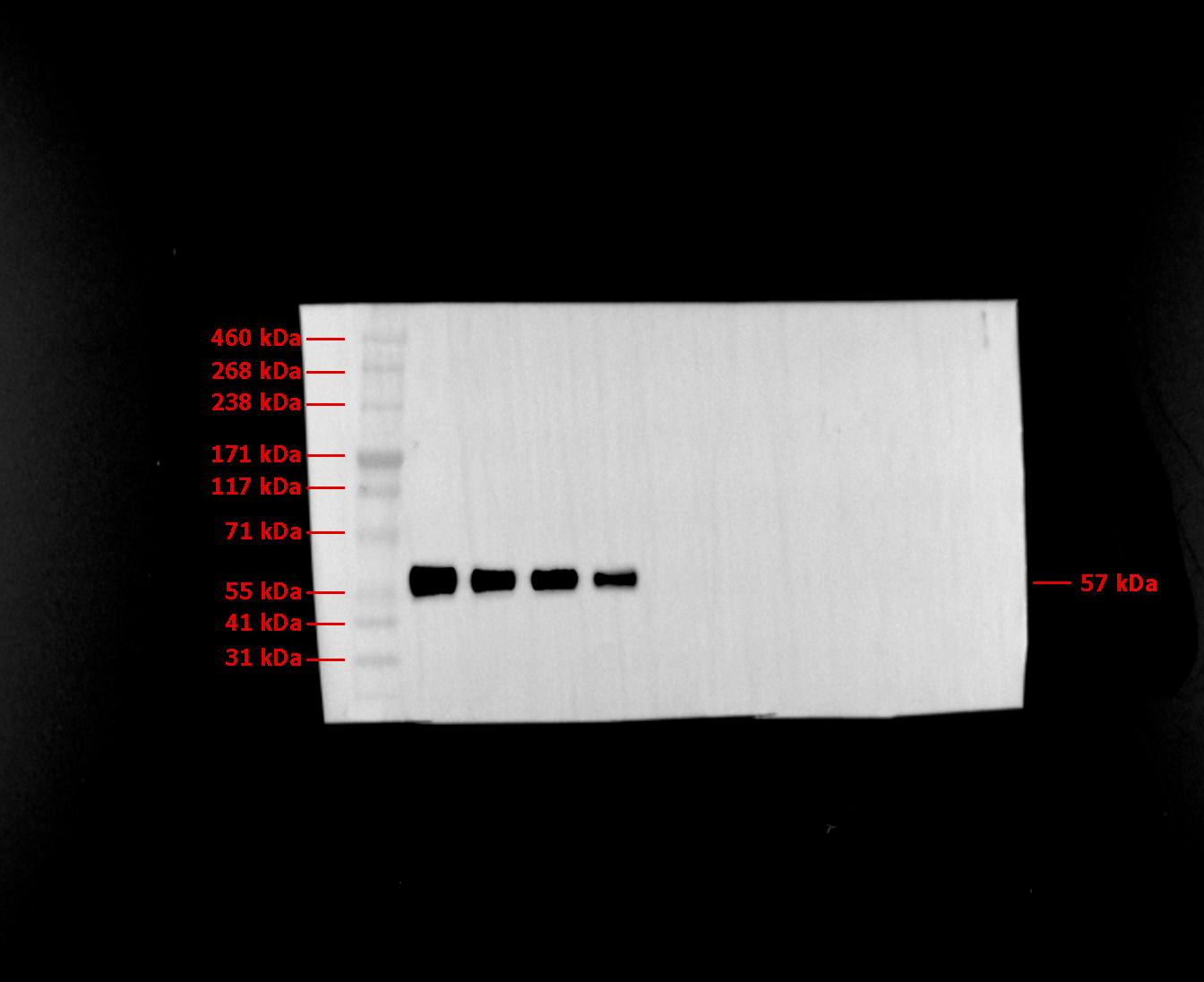

Supplement: Supplementary file 1 — Supplementary Material 1 [file 41065_2025_531_MOESM1_ESM.zip › original image for wb - marker/Original image Figure 2C/Figure 2C Vimentin.tif]

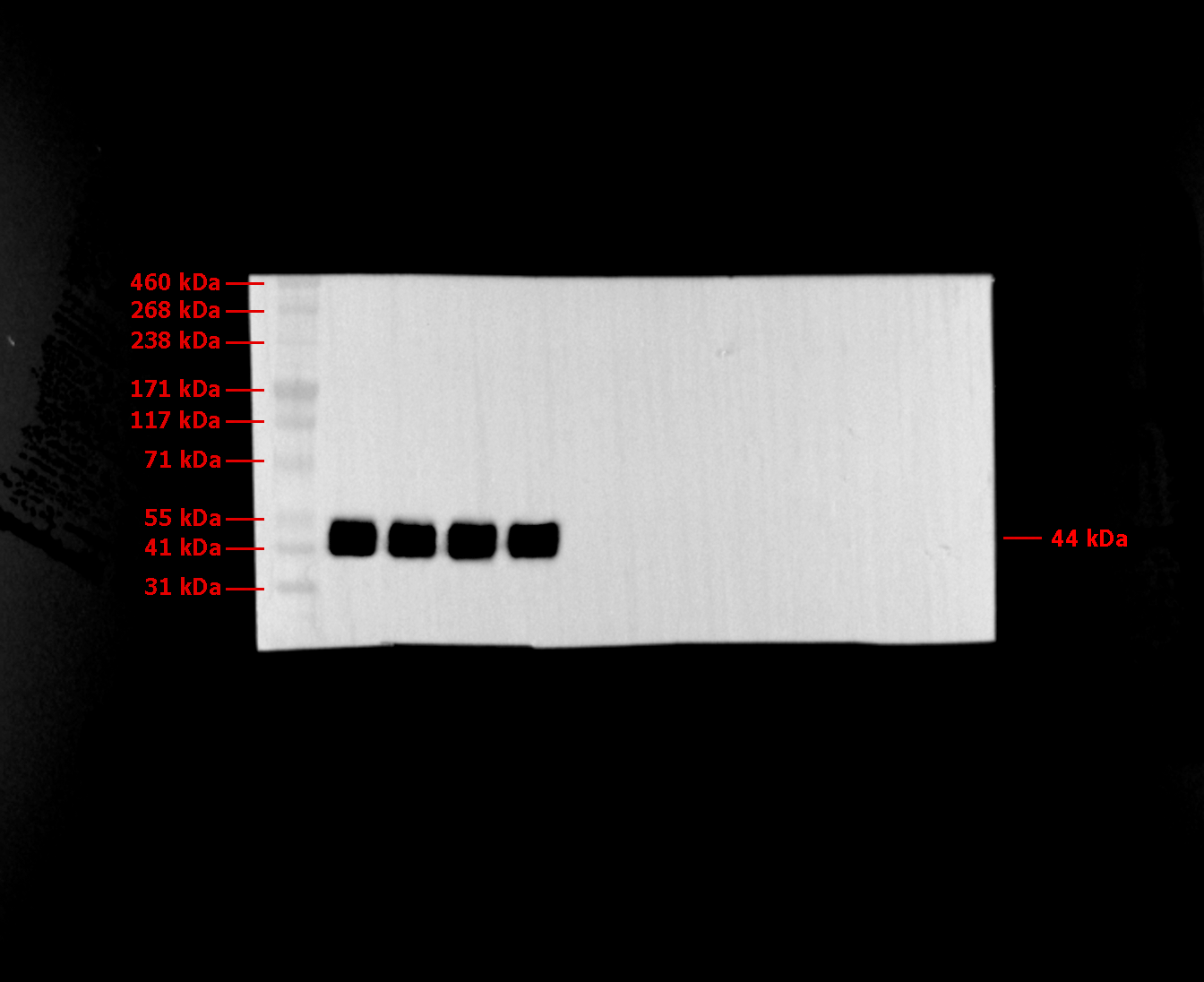

Supplement: Supplementary file 1 — Supplementary Material 1 [file 41065_2025_531_MOESM1_ESM.zip › original image for wb - marker/Original image Figure 2C/Figure 2C β-actin.tif]

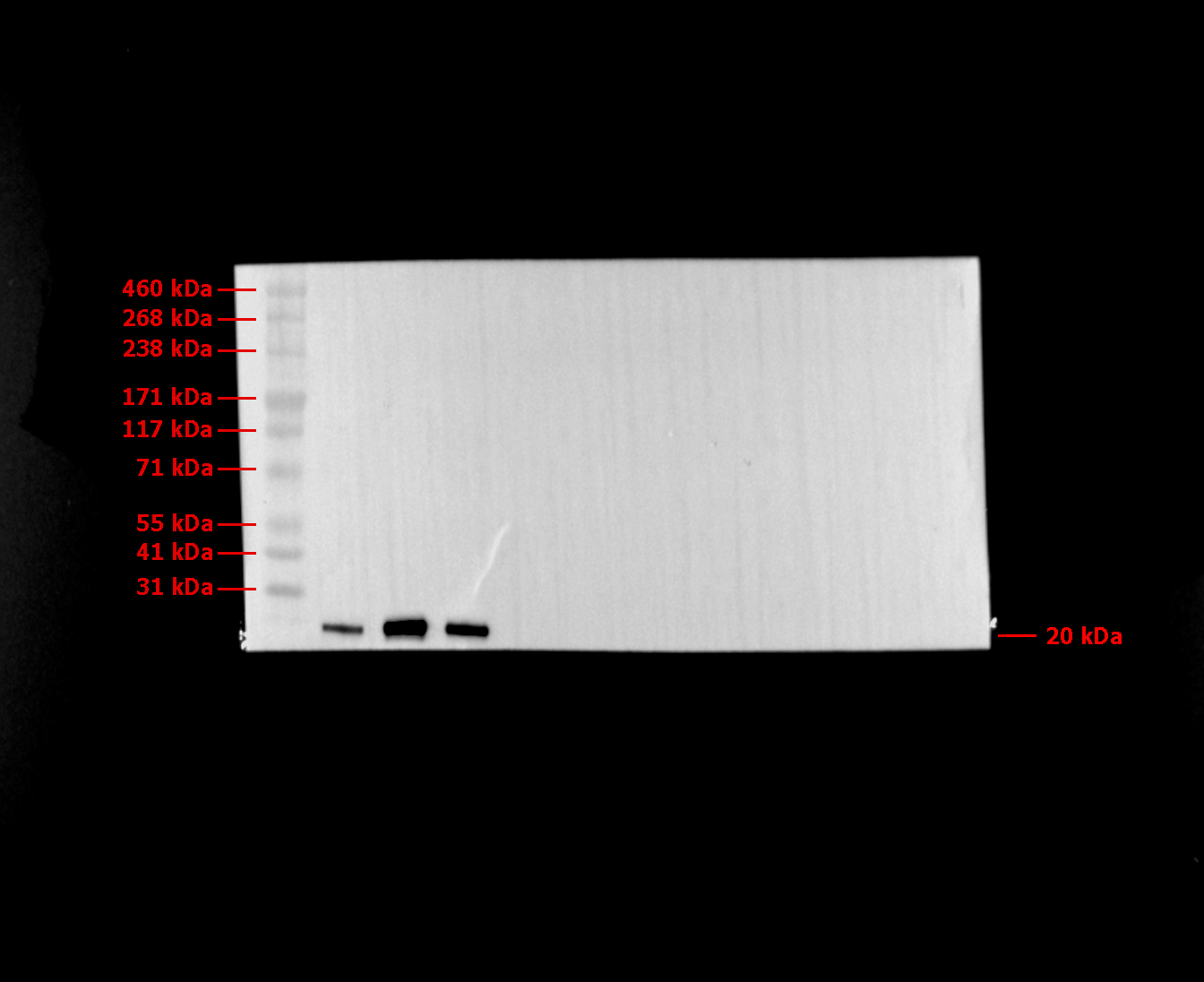

Supplement: Supplementary file 1 — Supplementary Material 1 [file 41065_2025_531_MOESM1_ESM.zip › original image for wb - marker/Original image Figure 5G/Figure 5G Bax.tif]

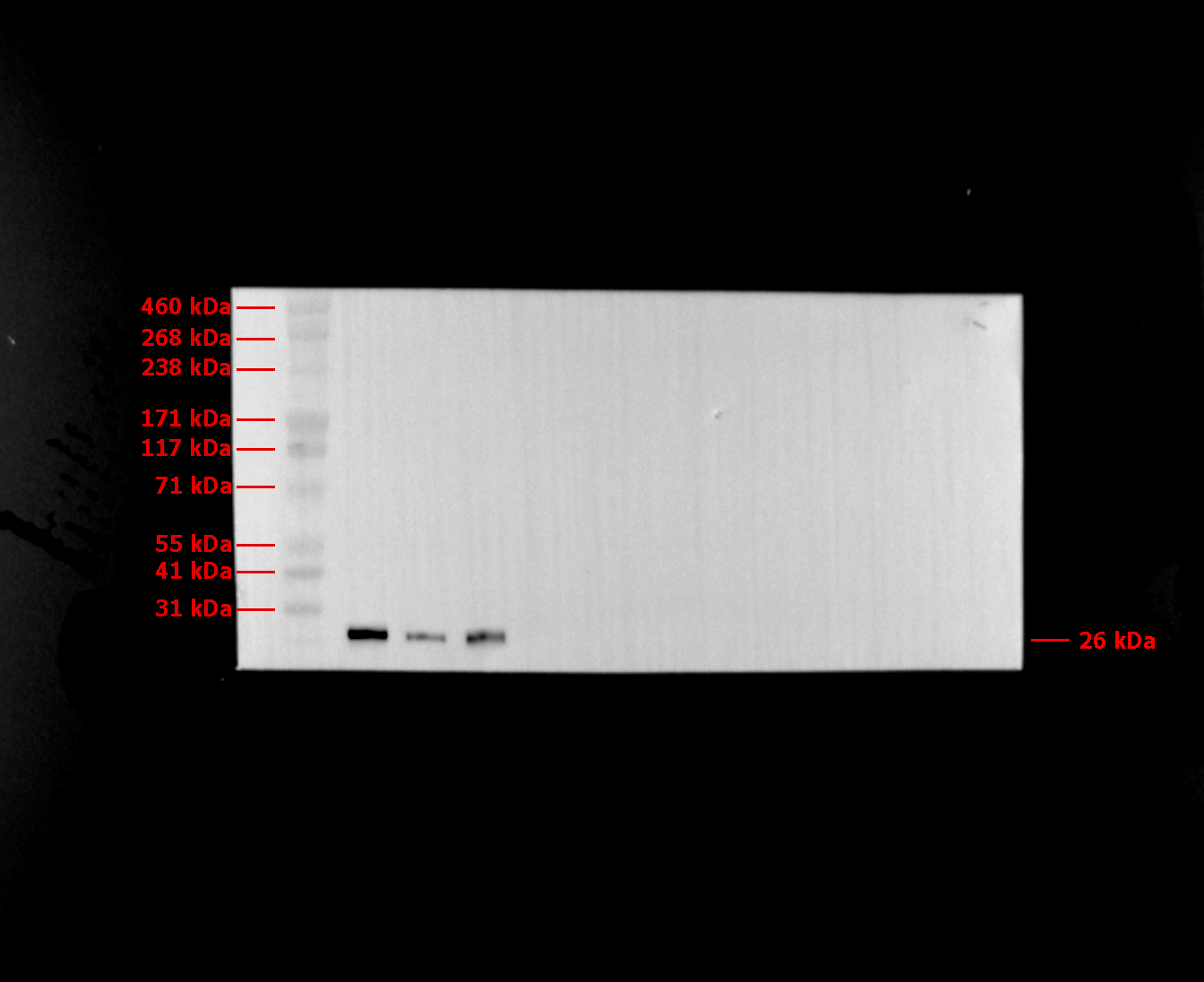

Supplement: Supplementary file 1 — Supplementary Material 1 [file 41065_2025_531_MOESM1_ESM.zip › original image for wb - marker/Original image Figure 5G/Figure 5G Bcl-2.tif]

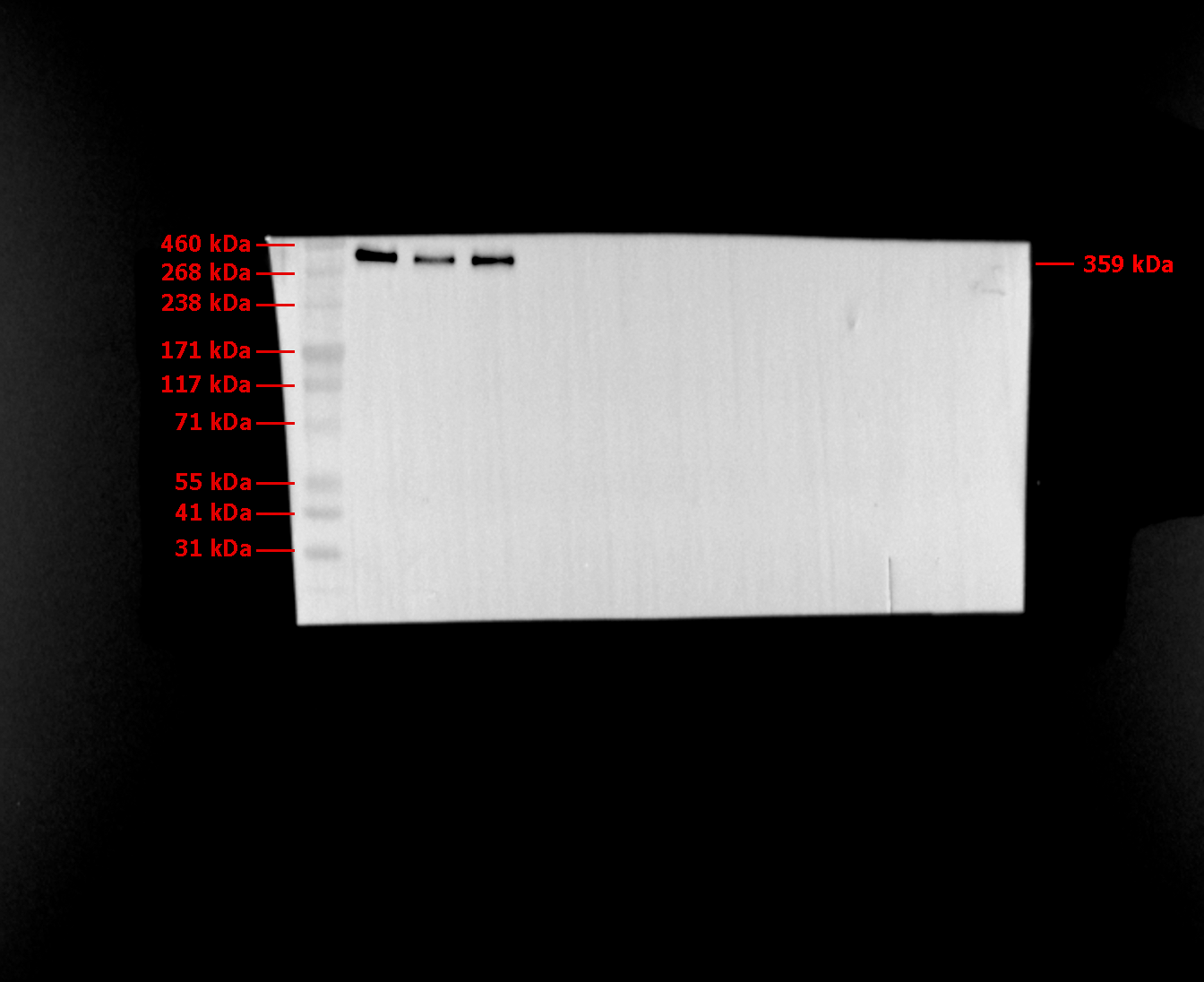

Supplement: Supplementary file 1 — Supplementary Material 1 [file 41065_2025_531_MOESM1_ESM.zip › original image for wb - marker/Original image Figure 5G/Figure 5G Ki-67.tif]

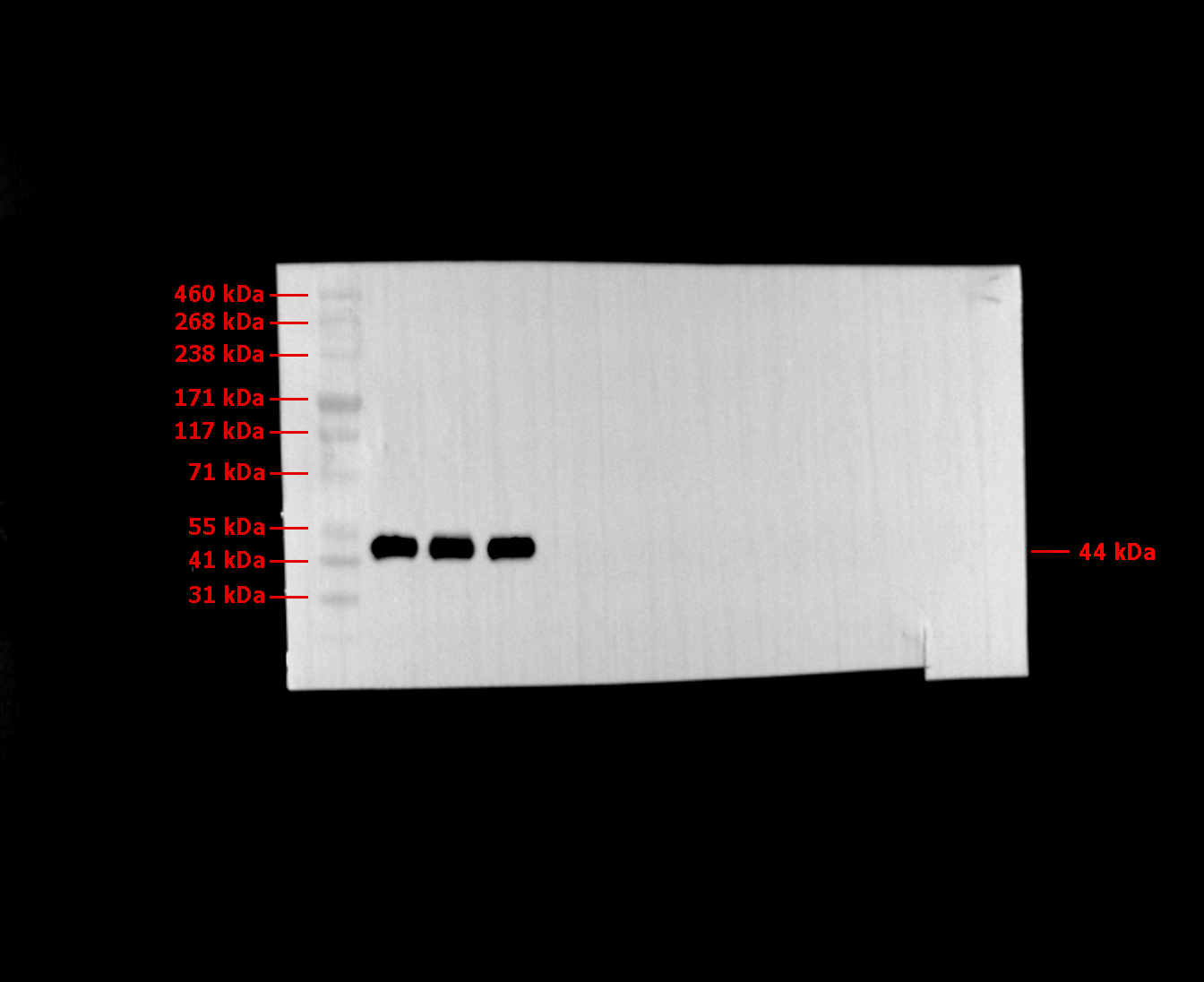

Supplement: Supplementary file 1 — Supplementary Material 1 [file 41065_2025_531_MOESM1_ESM.zip › original image for wb - marker/Original image Figure 5G/Figure 5G β-actin.tif]

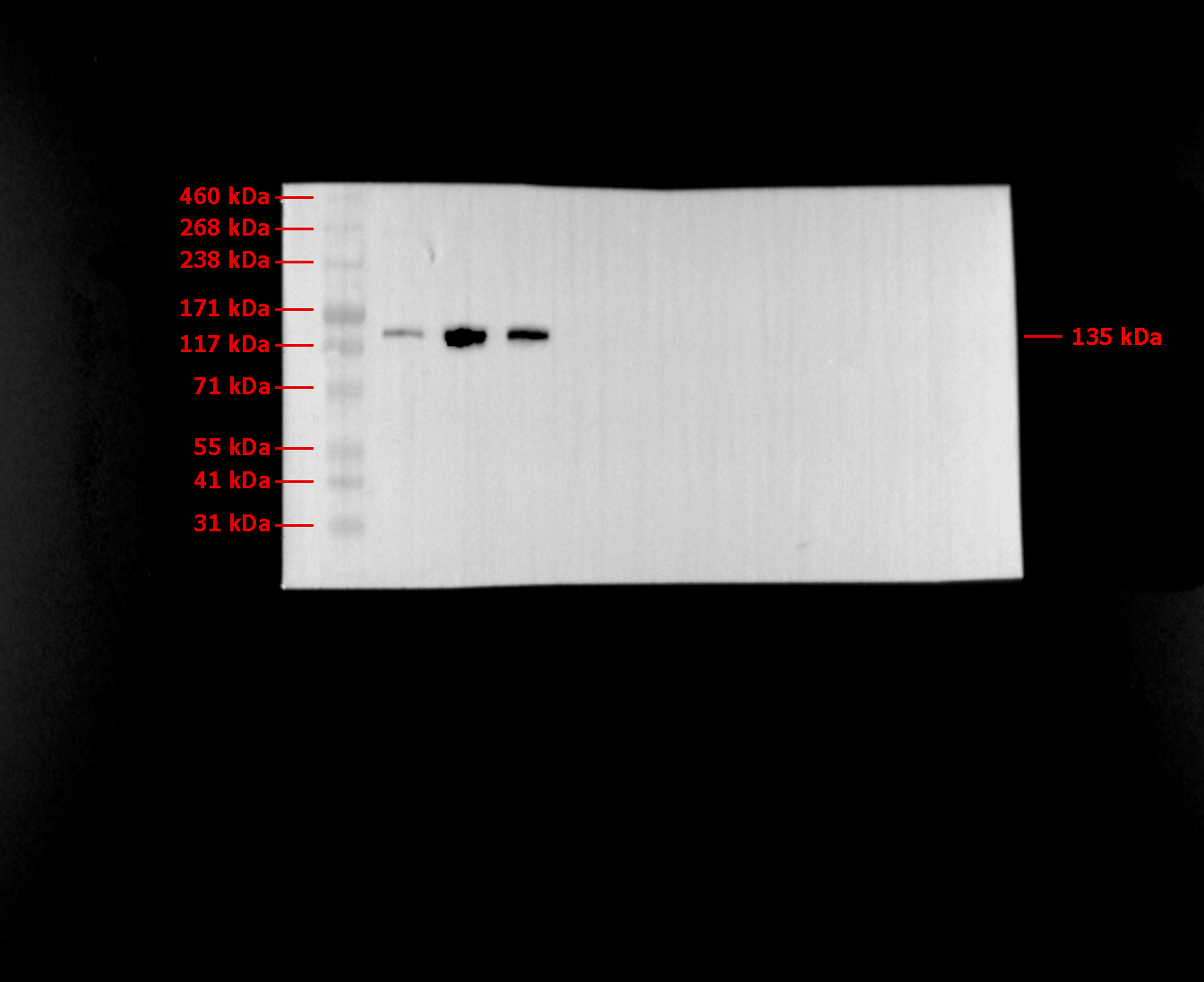

Supplement: Supplementary file 1 — Supplementary Material 1 [file 41065_2025_531_MOESM1_ESM.zip › original image for wb - marker/Original image Figure 5J/Figure 5J E-cadherin.tif]

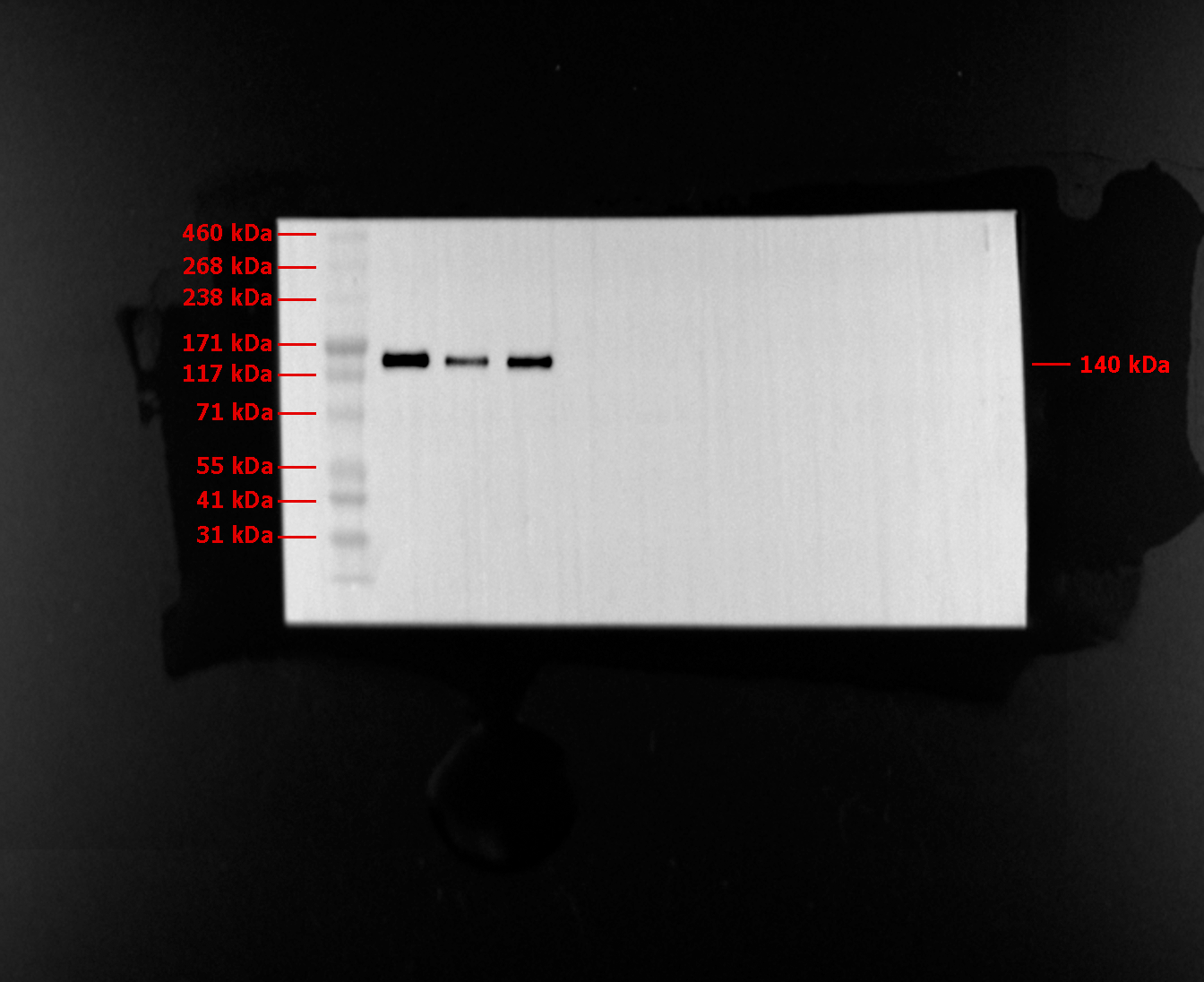

Supplement: Supplementary file 1 — Supplementary Material 1 [file 41065_2025_531_MOESM1_ESM.zip › original image for wb - marker/Original image Figure 5J/Figure 5J N-cadherin.tif]

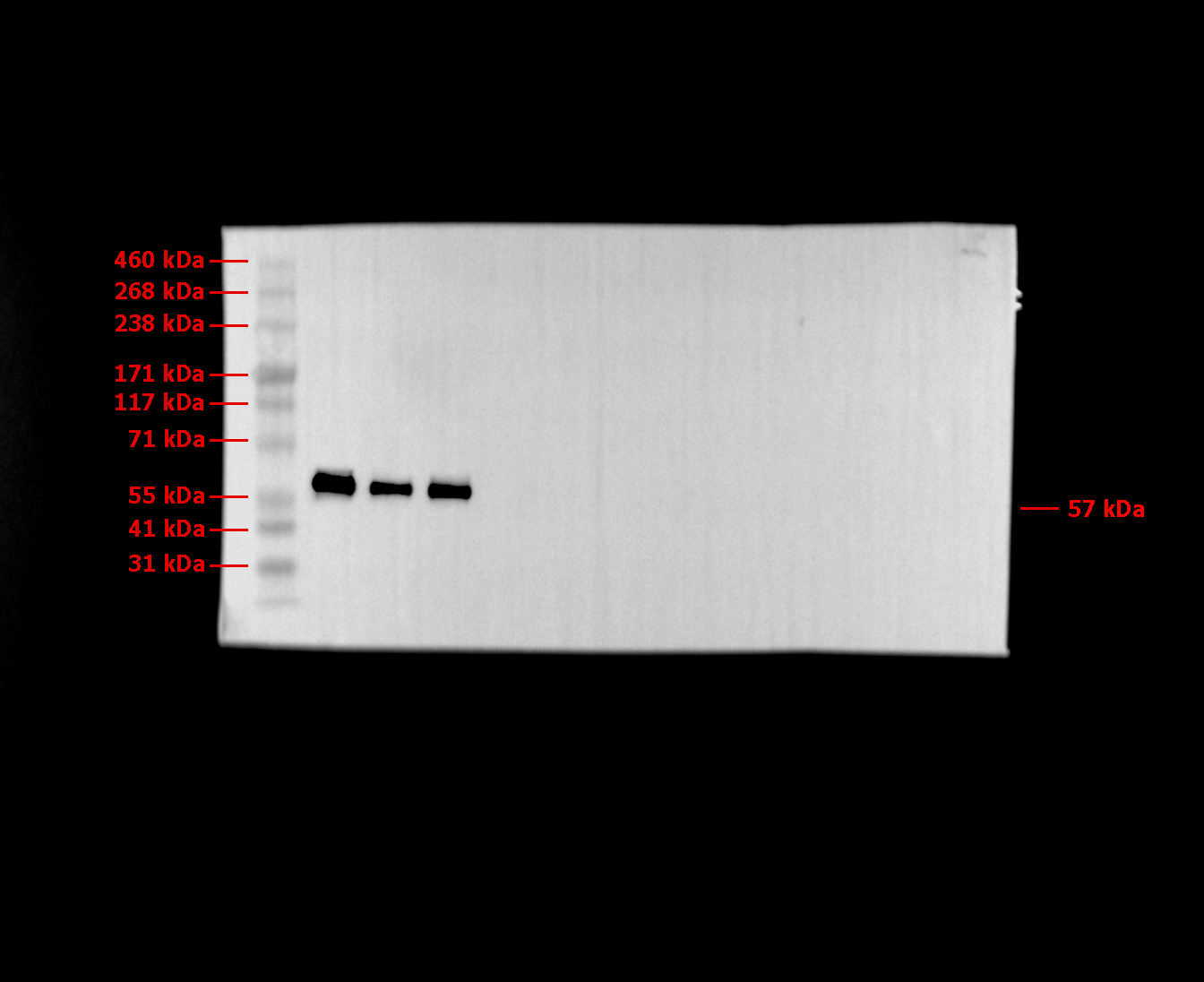

Supplement: Supplementary file 1 — Supplementary Material 1 [file 41065_2025_531_MOESM1_ESM.zip › original image for wb - marker/Original image Figure 5J/Figure 5J Vimentin.tif]

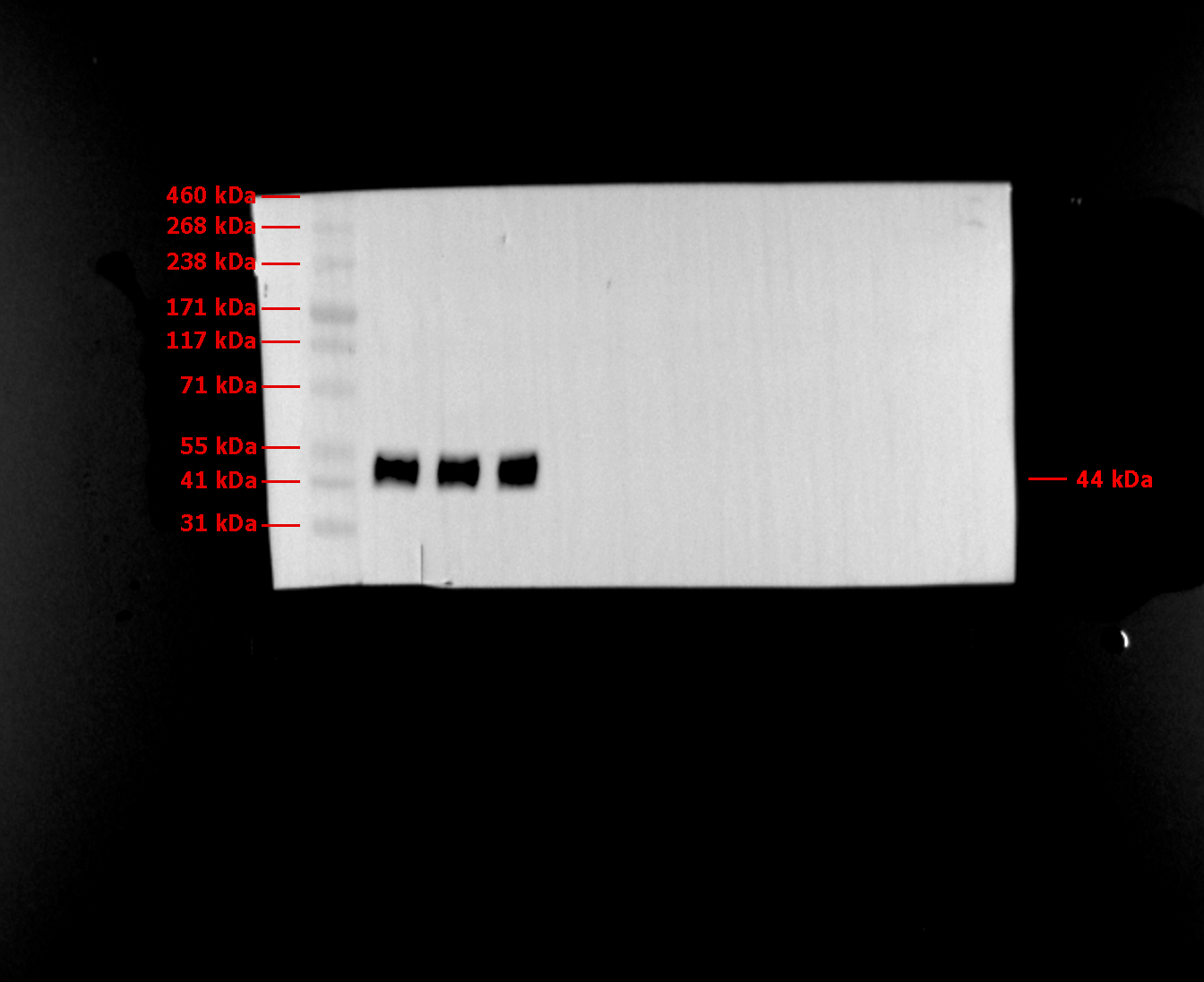

Supplement: Supplementary file 1 — Supplementary Material 1 [file 41065_2025_531_MOESM1_ESM.zip › original image for wb - marker/Original image Figure 5J/Figure 5J β-actin.tif]
